# The Mithralog EC-7072 Induces Chronic Lymphocytic Leukemia Cell Death by Targeting Tonic B-Cell Receptor Signaling

**DOI:** 10.3389/fimmu.2019.02455

**Published:** 2019-10-18

**Authors:** Seila Lorenzo-Herrero, Christian Sordo-Bahamonde, Gabriel Bretones, Ángel R. Payer, Ana P. González-Rodríguez, Esther González-García, Jhudit Pérez-Escuredo, Mónica Villa-Álvarez, Luz-Elena Núñez, Francisco Morís, Segundo Gonzalez, Alejandro López-Soto

**Affiliations:** ^1^Departamento de Biología Funcional, Inmunología, Universidad de Oviedo, Oviedo, Spain; ^2^Instituto Universitario de Oncología del Principado de Asturias, Oviedo, Spain; ^3^Instituto de Investigación Sanitaria del Principado de Asturias, Oviedo, Spain; ^4^EntreChem S.L., Oviedo, Spain; ^5^Departamento de Bioquímica y Biología Molecular, Universidad de Oviedo, Oviedo, Spain; ^6^Department of Hematology, Hospital Universitario Central de Asturias, Oviedo, Spain; ^7^Department of Hematology, Hospital de Cabueñes, Gijón, Spain

**Keywords:** CLL, apoptosis, BCR, mithralog, EC-7072

## Abstract

B-cell receptor (BCR)-dependent signaling is central for leukemia B-cell homeostasis, as underscored by the promising clinical results obtained in patients with chronic lymphocytic leukemia (CLL) treated with novel agents targeting components of this pathway. Herein, we demonstrate that the mithralog EC-7072 displays high *ex vivo* cytotoxic activity against leukemia cells from CLL patients independently from high-risk prognostic markers and IGHV mutational status. EC-7072 was significantly less toxic against T cells and NK cells and did not alter the production of the immune effector molecules IFN-γ and perforin. EC-7072 directly triggered caspase-3-dependent CLL cell apoptosis, which was not abrogated by microenvironment-derived factors that sustain leukemia cell survival. RNA-sequencing analyses revealed a dramatic EC-7072-driven reprograming of the transcriptome of CLL cells, including a wide downregulation of multiple components and targets of the BCR signaling pathway. Accordingly, we found decreased levels of phosphorylated signaling nodes downstream of the BCR. Crosslinking-mediated BCR activation antagonized CLL cell death triggered by EC-7072, increased the phosphorylation levels of the abovementioned signaling nodes and upregulated *BCL2* expression, suggesting that the mithralog disrupts CLL cell viability by targeting the BCR signaling axis at multiple levels. EC-7072 exerted similar or higher antileukemic activity than that of several available CLL therapies and displayed additive or synergistic interaction with these drugs in killing CLL cells. Overall, our findings provide rationale for future investigation to test whether EC-7072 may be a potential therapeutic option for patients with CLL and other B-cell malignancies.

## Introduction

Chronic lymphocytic leukemia (CLL), the most common leukemia in adults in western countries, is characterized by the progressive accumulation of mature-appearing clonal B cells expressing CD5, CD23, and CD19 surface markers in the blood, bone marrow and secondary lymphatic tissues. The risk of developing this malignancy increases with age and the median age of patients at diagnosis ranges from 65 to 70 years (1). While a subset of patients with CLL exhibit an indolent form of the disease, a progression of the malignancy is observed in a fraction of patients, who require therapeutic management (2). The heterogeneous behavior of CLL is, at least in part, underpinned by the presence of cytogenetic aberrations and the degree of somatic hypermutation in the immunoglobulin heavy-chain variable gene (IGHV), which bear great prognostic and therapeutic value (3). Thus, patients with unmutated IGHV typically suffer from an aggressive variant of CLL, while mutated IGHV correlates with a more indolent disease. Likewise, deletion of chromosome 17 del(17p) or chromosome 11 del(11q) are associated with poor therapeutic response and short survival (3). Whole-genome/exome sequencing studies have also unveiled heterogeneity within the malignancy (4–6) and recent epigenomic analyses uncovered distinct chromatin landscapes associated to clinical subtypes (7), altogether highlighting the complexity of CLL.

Management of patients with CLL comprises a wide range of therapeutic approaches, including chemotherapy (e.g., fludarabine, cyclophosphamide) or immunotherapy (e.g., anti-CD20 monoclonal antibodies) (8, 9). Nonetheless, response rates to currently available therapies in patients with CLL remain highly variable, mostly due to intrinsic or acquired resistance to treatment and drug toxicity, as exemplified by fludarabine-induced lymphocytopenia due to T cell depletion (8, 10). Genetic lesions, such as the abovementioned del(17p) and del(11q), or mutations in *NOTCH1* are key drivers of therapy resistance in patients with CLL, underscoring the need for novel treatments with a broader spectrum and safer effect independent of the cytogenetic profile of the patient. Currently, numerous novel treatments and combinations of approved drugs are being tested in clinical trials to increase the rates of complete remissions of the disease (8, 9).

The therapeutic armamentarium of patients with CLL has recently expanded toward molecularly targeted agents that inhibit key processes for leukemia cells (11). B-cell receptor (BCR) signaling stands out as a central player in this malignancy, since its aberrant activation provides growth and survival signals to leukemia cells (12, 13). The paramount relevance of BCR signaling to CLL homeostasis has prompted the development of novel inhibitors targeting BCR-related kinases, such as ibrutinib, a Bruton's tyrosine kinase (BTK) inhibitor with superior efficacy than several chemotherapy and chemoimmunotherapy treatments (9) [e.g., conventional therapy with bendamustine plus rituximab (12, 14)], or idelalisib, the first-in-class phosphatidylinositol 3-kinase delta (PI3Kδ) inhibitor for treatment of B-cell malignancies (15, 16). Along similar lines, the distinctive high levels of the antiapoptotic protein B-cell lymphoma 2 (BCL2) in CLL cells have opened a therapeutic window for molecules such as the recently FDA (Food and Drug Administration)-approved BCL2 antagonist venetoclax, which shows durable clinical activity in patients with relapsed or refractory disease when used alone or in combination with rituximab (17, 18). However, despite the clinical benefits demonstrated by these novel agents, a substantial fraction of patients eventually relapses owing to molecular mechanisms that confer resistance to targeted therapies, such as a point mutation in *BCL2* recently identified in patients with CLL refractory to treatment with venetoclax (19), which calls for the development of new therapeutic strategies for selected patients with CLL.

Over the years, antibiotics with antitumor properties have become part of the therapeutic arsenal in certain types of cancer. Particularly, mithramycin A (MTA) has been widely described as an extremely potent antitumor agent, owing to its DNA binding activity and the resulting inhibition of various transcription factors with essential roles in tumorigenesis (20). However, different studies have shown systemic toxicity and severe side effects associated to treatment with MTA, hence limiting its clinical use (21). To overcome this major problem, combinatorial biosynthesis has been applied to generate an array of analogs of MTA, so-called mithralogs, which frequently exhibit less toxicity and/or higher antitumor activity than MTA (22–26). Herein, we report that the mithralog EC-7072 (Mithramycin SK; MTM-SK) is highly cytotoxic *ex vivo* against circulating leukemia cells from patients with CLL. EC-7072 reprograms the transcriptome of primary CLL cells, resulting in a profound downregulation of multiple components of the BCR cascade. Consequently, CLL cells exposed to the mithralog exhibited hampered BCR-dependent signaling and activation of the BCR significantly antagonized EC-7072-driven CLL cell death. Noteworthy, EC-7072 showed comparable and additive or synergistic antileukemic activity with available targeted agents. Collectively, our studies suggest that EC-7072 may potentially constitute a novel and effective therapeutic option for patients with CLL.

## Materials and Methods

### Reagents

EC-7072 was provided by EntreChem S.L. (Oviedo, Spain). Stock solutions were prepared in dimethyl sulfoxide (DMSO) and stored at −80°C. DMSO was used as vehicle (control) in all experiments.

### Patient Samples

Blood samples from untreated patients with CLL (*n* = 63) were provided by Hospital Universitario Central de Asturias (Supplementary Table 1). Written informed consent was obtained from all the patients following the Declaration of Helsinki and samples were collected with approval from the local ethics committee (Comité de Ética de la Investigación del Principado de Asturias, case-19042016). CLL was diagnosed according to standard clinical and laboratory criteria. Buffy-coats from healthy donors (*n* = 20) were provided by Centro Comunitario de Sangre y Tejidos de Asturias. Peripheral blood mononuclear cells (PBMCs) were isolated by Ficoll gradient centrifugation from healthy donors and patients with CLL and used fresh. For some experiments, leukemia cells were isolated by using EasySep^TM^ Direct Human B-CLL Cell Isolation Kit (Stemcell Technologies) and T cells were purified using Pan T Cell Isolation Kit (Miltenyi Biotec), following the manufacturer's recommendations. Purity of isolated populations was determined by flow cytometry and only samples with purity higher than 90% were employed. PBMCs and isolated immune subsets were cultured in RPMI 1640 (Lonza) supplemented with 10% heat-inactivated fetal bovine serum (FBS) (Sigma-Aldrich), 1 mM sodium pyruvate, 2 mM L-glutamine, 100 U/mL penicillin and 10 μg/mL streptomycin at 37°C and 5% CO_2_.

### Immune Cell Subset Identification

To determine immune cell populations, the following antibodies were employed: anti-CD19-APC, anti-CD3-APC, anti-CD4-PerCP, and anti-CD8-APC (all from Immunostep); anti-CD3-FITC and anti-CD56-APC (Cytognos); and anti-CD3-PE/Cy7 and anti-CD5-APC/Cy7 (Biolegend). The subsets of immune cells were identified as follows: T cells were defined as CD3^+^CD56^−^, NK cells were identified as CD3^−^CD56^+^ and healthy B cells as CD19^+^. Leukemia cells were defined as CD19^+^, as the percentage of healthy B cells (CD19^+^CD5^−^) detected in PBMCs from patients with CLL was <2% (data not shown). Additionally, antibodies from Biolegend were used to detect surface expression of CD79A (clone: HM47), CD79B (clone: CB3-1), and IgM (clone: MHM-88). Cells were analyzed in a BD FACS Canto II flow cytometer with FACS Diva software (Beckton Dickinson).

### Absolute Cell Number Quantification

To evaluate absolute cell numbers, an equal volume of PKH26 reference microbeads (Sigma-Aldrich) was added to each sample after staining for immune subset identification. Microbeads (5 × 10^3^) were acquired by flow cytometry and absolute numbers of each immune population within the sample were calculated. Normalized percentage values were calculated considering the control (DMSO)-treated cells as 100%.

### Detection of Apoptosis

Cell viability and apoptosis were evaluated by double-staining with the mitochondrial inner transmembrane potential (ΔΨ_m_)-sensitive dye DiOC_6_(3) (3,3′-dihexyloxacarbocyanine iodide; Sigma-Aldrich) and the exclusion dye propidium iodide (PI; Immunostep), allowing identification of apoptotic [DiOC_6_(3) low PI^−^] and dead [PI^+^] cells (27, 28). Briefly, PBMCs were resuspended in culture medium containing 20 nM DiOC_6_(3) and incubated for 30 min at 37°C. Then, PI (1 μg/mL) was added and cells were further incubated for 10 min at room temperature (RT). Cells were analyzed by flow cytometry considering DiOC_6_(3)^+^PI^−^ cells as viable. In some experiments, apoptosis was evaluated by staining with Annexin V/PI. Thus, PBMCs were incubated in Annexin V binding buffer containing 5 μg/mL Annexin V-FITC (Biolegend) for 15 min at RT and PI (1 μg/mL) was subsequently added. Data were normalized to the untreated control as a percentage. Only samples with at least 60% of viable cells (DiOC_6_(3)^+^; Annexin V^−^) in the control (DMSO) condition were employed in this study.

### Caspase-3 Activity Assay

Intracellular levels of activated caspase-3 were measured by using CaspGLOW ™ Fluorescein Active Caspase-3 Staining Kit (Invitrogen). In brief, EC-7072 (200 nM)- or DMSO-treated PBMCs from patients with CLL were incubated with FITC-DEVD-fmk for 30 min at 37°C and fluorescence was next analyzed by flow cytometry in leukemia cells. For some experiments, cells were pretreated for 2 h with the indicated doses of the broad-spectrum caspase inhibitor Z-VAD-fmk (Selleckchem).

### RNA-Seq Analysis

Isolated leukemia cells from 8 patients with CLL were incubated with 200 nM EC-7072 or DMSO for 6 h. Total RNA from isolated CLL cells was extracted using RNeasy Mini-Kit (Qiagen). RNA integrity and concentration were determined using an Agilent-2100 Bioanalyzer (Agilent Technologies) and high-quality RNA samples were further processed. RNA-seq libraries were prepared using TruSeq-Stranded mRNA LT Sample Prep Kit (Illumina) and checked for quality using a DNA 1,000 chip on a 2,100 Bioanalyzer. Each library was sequenced on a HiSeq 4,000 (Illumina) to generate ~20 million uniquely-mapping reads per sample. FASTQ files were first evaluated using FastQC for quality control checks (http://www.bioinformatics.babraham.ac.uk/projects/fastqc/). No problems or biases associated to library preparation or sequencing were identified. Transcript abundance was directly quantified with Salmon (29), employing the human transcriptome version GRCh38 (Ensembl) as reference. Transcript-level quantifications were aggregated for gene-level differential analysis with DESeq2 (30), applying a multifactor design for paired samples. Finally, gene set enrichment analysis was carried out with PathfindR (31). Further gene analysis and representations were performed excluding genes with mean expression lower than 25 in control (DMSO) and EC-7072 conditions.

The RNA-seq datasets generated for this study have been deposited in the Gene Expression Omnibus database (GEO; https://www.ncbi.nlm.nih.gov/geo/) under accession number GSE123777.

### Phosphoflow

Protein phosphorylation levels were evaluated by phosphoflow. Briefly, isolated leukemia cells from patients with CLL were treated with 200 nM EC-7072 or DMSO for 8 or 12 h. In some experiments, BCR signaling was activated with goat F(ab')2 anti-IgM antibody (10 μg/mL; SouthernBiotech) for 45 min. Right afterwards, cells were fixed with 1.8% paraformaldehyde for 10 min at RT and permeabilized with 90% ice-cold methanol for 25 min at 4°C. Finally, cells were incubated with specific phospho-antibodies (Supplementary Table 2) for 30 min at RT and mean fluorescence intensity (MFI) was analyzed by flow cytometry.

### Western Blotting

Isolated CLL cells were treated with 200 nM EC-7072 for 3, 8, and 12 h and lysed with lysis buffer [50 mM Tris-HCl (pH 8), 150 mM NaCl, 1 mM EDTA, 1% NP-40, 20 mM NaF, EDTA-free protease inhibitor cocktail and phosSTOP™ (Roche)]. After heat denaturation, 25 μg protein were resolved on 10 and 15% SDS-PAGE gels and transferred to PVDF membranes (Sigma-Aldrich). Membranes were blocked with 5% BSA and incubated overnight at 4°C with the corresponding primary antibodies (Supplementary Table 3). Blots were developed with secondary antibodies conjugated to IRDye680 or IRDye800 (Li-Cor Biosciences) and signal was detected on an Odyssey scanner.

### Statistical Analysis

Sample size was based on the number of patients or volunteers enrolled to previously designed studies. Statistical analyses were performed using GraphPad Prism 6 software (GraphPad Software Inc.) and appropriate statistical tests were applied to each experiment. For all tests, differences were considered statistically significant for *p*-values: *P* < 0.05 (^*^), *P* < 0.01 (^**^), *P* < 0.001 (^***^).

For drug combination studies, synergy was evaluated using CalcuSyn Version 2.0 software (Biosoft) that allows the calculation of the combination index (CI) based on the algorithm reported by Chou and Talalay (32). CI values <0.8 represent synergistic effect, values between 0.8 and 1.2 indicate additive effect and values > 1.2 represent an antagonistic effect (32, 33). CI values for non-fixed ratio combinations from independent experiments were generated and plotted.

For further information on experimental protocols employed in this study, please refer to Supplementary Methods.

## Results

### EC-7072 Is Highly Effective Against Primary Leukemia Cells From Patients With CLL Independently From Cytogenetic Aberrations and IGHV Mutational Status

**Figure 1:**
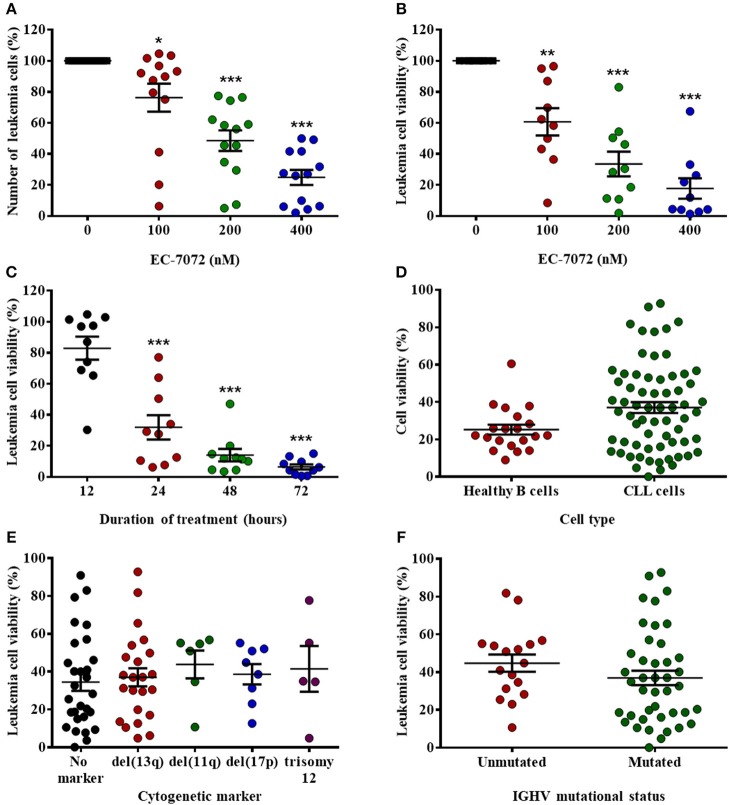
EC-7072 has a cytotoxic effect against primary leukemia cells from patients with CLL. **(A)** PBMCs from patients with CLL (*n* = 13) were incubated with increasing concentrations of EC-7072 (0–400 nM) for 24 h. Numbers of leukemia cells were evaluated by flow cytometry. The graph represents the number of cells normalized to their respective control (DMSO) condition for each individual patient. **(B)** PBMCs from patients with CLL (*n* = 10) were incubated with or without EC-7072 (100–400 nM) for 24 h. Viability of leukemia cells was determined by cytofluorometric assessment of DiOC_6_(3)/PI staining. **(C)** PBMCs from patients with CLL (*n* = 10) were incubated with or without EC-7072 (200 nM) for 12 to 72 h. Viability of leukemia cells was determined by DiOC_6_(3)/PI staining. **(D)** PBMCs from patients with CLL (*n* = 63) or healthy donors (*n* = 20) were incubated with or without EC-7072 (200 nM) for 24 h. Viability of leukemia cells and healthy B cells was evaluated by DiOC_6_(3)/PI staining. Graph represents the percentage of viable [DiOC_6_(3)^+^] cells normalized to their respective control condition. **(E)** Viability of leukemia cells was evaluated by DiOC_6_(3)/PI staining in PBMCs from patients with CLL (*n* = 62) incubated with EC-7072 (200 nM) for 24 h. Cytogenetic profile was obtained for each patient. **(F)** Viability of leukemia cells was evaluated by DiOC_6_(3)/PI staining in PBMCs from patients with CLL (*n* = 59) incubated with EC-7072 (200 nM) for 24 h. Mutational status of IGHV was determined for each patient. Graphs depict the percentage of viable [DiOC_6_(3)^+^] cells normalized to their respective control (DMSO) condition for each individual patient. Dark lines represent mean ± standard error of the mean (SEM) (^*^P < 0.05; ^**^P < 0.01; ^***^P < 0.001; Student's *t*-test).

EC-7072 is a mithralog generated by targeted inactivation of a ketoreductase implicated in the biosynthesis of MTA (22) (Supplementary Figure 1A). To evaluate the effect of the compound in CLL, we assessed the *in vitro* cytotoxic activity of EC-7072 against primary CLL cells. Treatment of PBMCs isolated from CLL patients with increasing concentrations of EC-7072 significantly decreased leukemia cell numbers and viability in a dose- (Figures 1A,B and Supplementary Figure 1B) and a time- (Figure 1C) dependent manner. The antileukemic activity of EC-7072 was comparable to that of MTA (Supplementary Figure 1C). However, in sharp contrast with the high toxicity of MTA, the mithralog did not markedly affect the viability of non-tumoral cells such as primary human fibroblasts and an immortalized cell line derived from normal adult human kidney (HK-2) (Supplementary Figures 2A,B). Nonetheless, EC-7072 triggered death of normal B cells from healthy volunteers, which, in addition to its lethal effect on CLL cells, suggests that the compound displays high cytotoxicity against B cells (Figure 1D). In agreement, EC-7072 significantly reduced the surviving cell fraction of a panel of tumor cell lines derived from B-cell hematologic malignancies (Supplementary Figure 2C).

Next, we investigated whether the cytotoxic effect of EC-7072 was affected by the presence of chromosomal and molecular genetic alterations that are markers of patient prognosis and therapy response in CLL (34). Noteworthy, no differences in the sensitivity to EC-7072 were observed among patients carrying diverse cytogenetic aberrations that define high-risk CLL, including those mediating apoptotic responses to genotoxic compounds, such as del(17p) or del(11q) (Figure 1E). Likewise, the mutational status of IGHV did not significantly affect the sensitivity to EC-7072 in patients with CLL (Figure 1F). Overall, these findings support that the mithralog EC-7072 exerts a strong *ex vivo* antileukemic activity against primary cells from patients with CLL, including those bearing genetic markers typically associated to clinical resistance to current chemotherapy modalities.

### EC-7072 Does Not Markedly Alter the Homeostasis of Healthy Immune Cells From Patients With CLL

**Figure 2:**
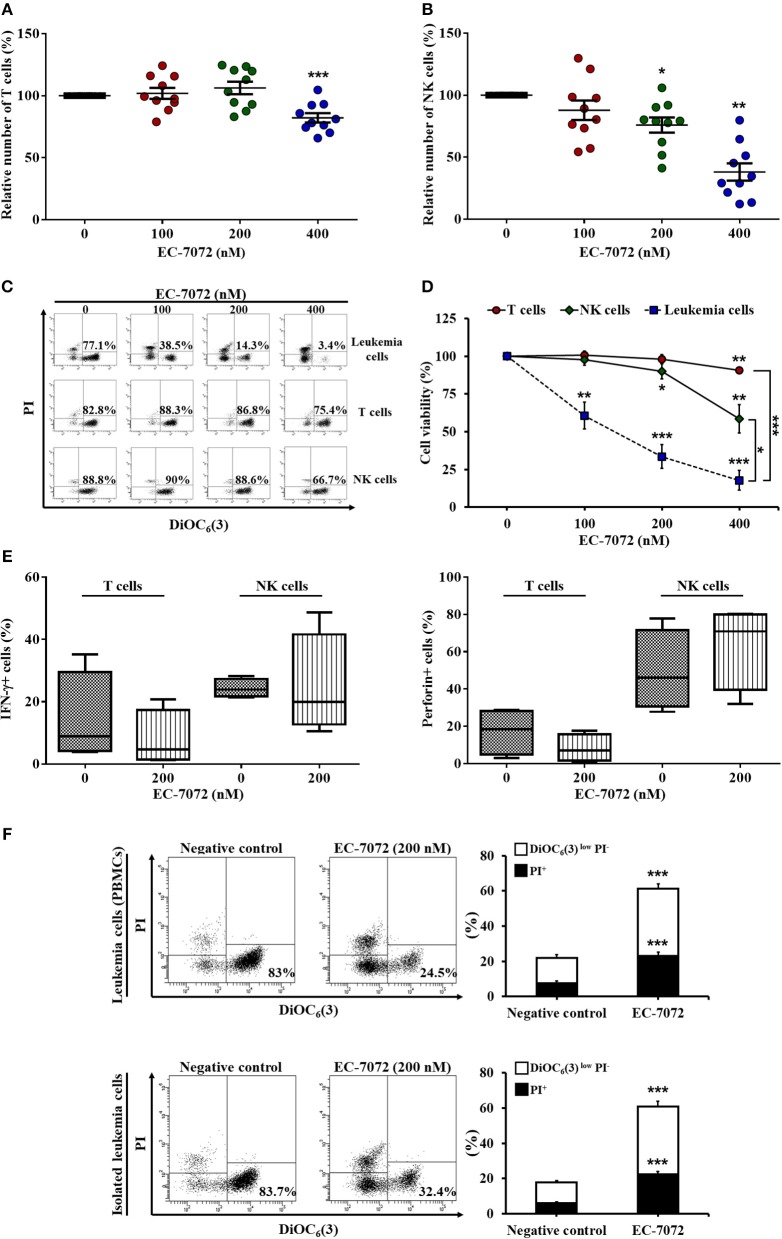
EC-7072 shows reduced toxicity on healthy immune subsets from patients with CLL. PBMCs from patients with CLL (*n* = 10) were incubated with increasing concentrations of EC-7072 (0–400 nM) for 24 h. Numbers of T cells (CD3^+^CD56^−^) **(A)** and NK cells (CD3^−^CD56^+^) **(B)** were evaluated by flow cytometry. Graphs represent the number of cells normalized to their respective control (DMSO) condition for each individual patient and dark lines correspond to mean ± SEM. **(C,D)** PBMCs from patients with CLL (*n* = 6–10) were incubated with increasing concentrations of EC-7072 (0–400 nM) for 24 h and viability of immune subsets (leukemia cells, T cells, and NK cells) was determined by cytofluorometric assessment of DiOC_6_(3)/PI staining. Dot plots depict a representative patient with CLL and percentages within refer to viable [DiOC_6_(3)^+^] cells **(C)**. Graphs represent the percentage of viable [DiOC_6_(3)^+^] cells normalized to their respective control (DMSO) condition **(D)**. **(E)** IFN-γ and perforin expression upon PMA/ionomycin stimulation was evaluated in T cells and NK cells by intracellular flow cytometry in PBMCs from patients with CLL (*n* = 4) exposed to EC-7072 (200 nM) for 24 h. The graph depicts the percentage of IFN-γ^+^ or perforin^+^ cells, respectively. **(F)** PBMCs and isolated leukemia cells from patients with CLL (*n* = 24) were treated with EC-7072 (200 nM) for 24 h and apoptosis was examined by DiOC_6_(3)/PI staining. Dot plots depict a representative patient and percentages within refer to viable [DiOC_6_(3)^+^] cells. Bars represent the percentages of apoptotic [DiOC_6_(3)^low^ PI^−^] and dead [PI^+^] cells (mean ± SEM) (^*^P < 0.05; ^**^P < 0.01; ^***^P < 0.001; Student's *t*-test).

Based on our results showing the efficacy of EC-7072 to target B cells, we next assessed the effect of the compound toward healthy immune subsets, T lymphocytes and natural killer (NK) cells, which are chronically exposed to leukemia cells in the peripheral blood of patients with CLL. Treatment of PBMCs from patients with CLL with increasing doses of EC-7072 exerted negligible effects on T cell numbers (Figure 2A) and viability (Figures 2C,D), finding no differences between CD4^+^ and CD8^+^ T cell subpopulations (Supplementary Figure 3). Likewise, NK cell survival was only slightly altered by exposure to 200 nM EC-7072, a concentration of the mithralog that dramatically induced CLL cell death (Figure 1B); and even high doses (400 nM) of the compound were significantly less toxic against NK cells than leukemia cells (Figures 2B–D). We thus performed all subsequent experiments with 200 nM EC-7072, unless otherwise indicated. Exposure of PBMCs from healthy donors to EC-7072 rendered comparable effects on cell numbers and viability of T lymphocytes and NK cells, reinforcing the notion that the compound is clearly less toxic against these immune subsets (Supplementary Figure 4).

Remarkably, the production of the key antitumor effector molecules interferon (IFN)-γ and perforin by T cells and NK cells was not significantly altered upon treatment with the mithralog, suggesting that the antitumor function of immune effector cells is not impaired by EC-7072 (Figure 2E). Thus, to test whether the observed leukemia cell death induced by this compound was mediated by the immune cells present in the environment of peripheral CLL cells, we evaluated the effect of EC-7072 on isolated leukemia cells from patients with CLL. As shown in Figure 2F, the potent antileukemic activity of EC-7072 on PBMCs from patients with CLL was mimicked when isolated leukemia cells were used, demonstrating that the mithralog exerts its cytotoxic effect on leukemia cells independently of the activity of immune effector cells.

### EC-7072 Induces Caspase-Dependent Apoptosis in Primary CLL Cells That Is Not Counteracted by Microenvironmental Supportive Stimuli

**Figure 3:**
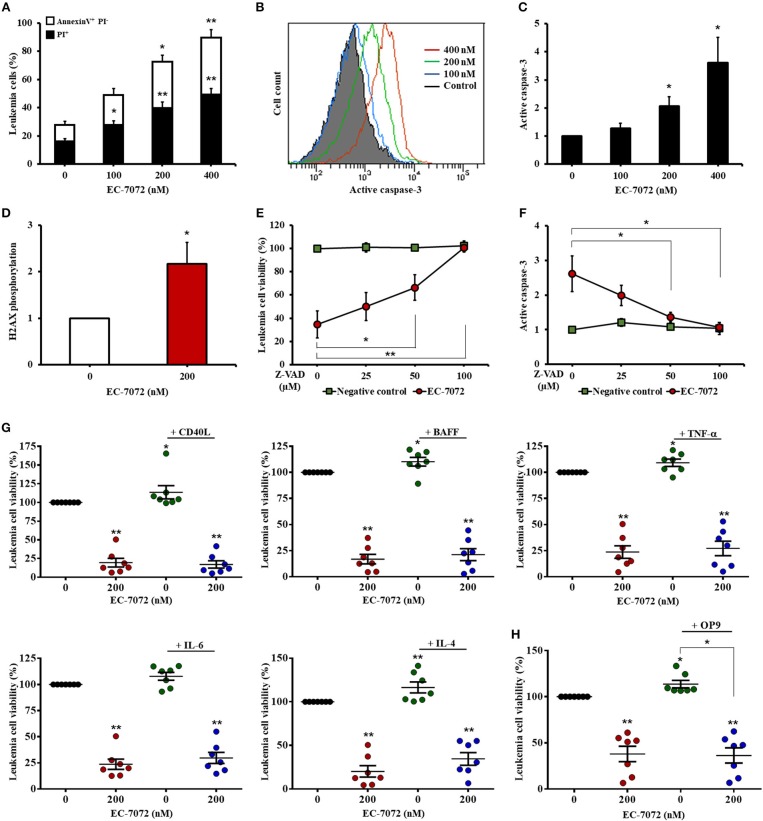
EC-7072 induces caspase-dependent apoptosis on primary CLL cells. **(A)** Apoptosis of leukemia cells was evaluated by cytofluorometric assessment of Annexin V/PI staining in PBMCs from patients with CLL (*n* = 6) treated with EC-7072 (0–400 nM) for 24 h. Bars represent the percentages of apoptotic [Annexin V^+^ PI^−^] and dead [PI^+^] cells. **(B,C)** Levels of active caspase-3 in leukemia cells were assessed by flow cytometry in PBMCs from patients with CLL (*n* = 6) treated with EC-7072 (0–400 nM) for 24 h. Histograms illustrate the MFI of a representative patient **(B)**. Bars depict the MFI normalized to the control (DMSO) condition **(C)**. **(D)** Levels of phosphorylated H2AX were assessed by phosphoflow in isolated leukemia cells from patients with CLL (*n* = 4) treated with 200 nM EC-7072 for 12 h. Bars depict the MFI normalized to the control (DMSO) condition. **(E,F)** PBMCs from patients with CLL (*n* = 6) were pretreated with increasing doses of Z-VAD-fmk (0–100 μM) followed by exposure to EC-7072 (200 nM) for 24 h. **(E)** Viability of leukemia cells was evaluated by DiOC_6_(3)/PI staining. The graph depicts the percentage of viable [DiOC_6_(3)^+^] cells normalized to the control (DMSO) condition. **(F)** Levels of active caspase-3 were examined by flow cytometry in leukemia cells. The graph depicts the MFI normalized to the control (DMSO) condition. **(G)** PBMCs from patients with CLL (*n* = 7) were incubated with 200 nM EC-7072 in combination with 100 ng/mL CD40L, 50 ng/ml BAFF, 40 ng/mL TNF-α, 40 ng/mL IL-6 or 40 ng/mL IL-4 for 24 h. Leukemia cell viability was assessed by DiOC_6_(3)/PI staining. Graphs depict the mean percentage of viable [DiOC_6_(3)^+^] cells normalized to their respective control (DMSO) condition for each individual experiment. **(H)** PBMCs from patients with CLL (*n* = 7) were co-cultured with OP9 cells for 72 h and were exposed to 200 nM EC-7072 for the last 24 h. Viability of leukemia cells was evaluated by DiOC6(3)/PI staining. Graphs depict the percentage of viable [DiOC6(3)^+^] cells normalized to their respective control (DMSO) condition for each individual patient. (Mean ± SEM) (^*^P < 0.05; ^**^P < 0.01; Student's *t*-test).

We next sought to gain further insight into the cytotoxic activity of EC-7072 against CLL cells. Our experiments performed with DiOC_6_(3)/PI evinced a compromised leukemia cell plasma membrane integrity and mitochondrial transmembrane potential, which are apoptosis-related parameters, upon EC-7072 treatment. These results were further verified by Annexin V/PI flow cytometric analyses showing that EC-7072 significantly induces CLL cell apoptosis (Figure 3A). Conversely, no significant apoptosis was detected in T cells isolated from patients with CLL cultured in the presence of EC-7072, further supporting the notion that the mithralog displays selective toxicity against B cells compared to other immune cells (Supplementary Figure 5).

Treatment of CLL cells with EC-7072 resulted in a dose-dependent increase of the intracellular levels of active caspase-3 (Figures 3B,C) and heightened phosphorylation of histone H2AX (Figure 3D), which has been reported to constitute a cellular response triggered by caspase activation during apoptosis (35), suggesting that EC-7072 may promote leukemia cell death through a caspase-dependent pathway. Indeed, treatment with increasing doses of the broad-spectrum caspase inhibitor Z-VAD-fmk completely abrogated the death of primary CLL cells induced by the mithralog (Figure 3E). As expected, the protective effect of Z-VAD-fmk correlated with a decrease of active caspase-3 levels on leukemia cells, thus reinforcing the specificity of the assay (Figure 3F).

A hallmark of CLL is the strong dependence of leukemia cells for prosurvival and growth signals provided by the microenvironment (36). Thus, we next explored whether exposure to CD40 ligand (CD40L), B-cell activating factor (BAFF), tumor necrosis factor (TNF)-α, interleukin (IL)-6 or IL-4, which are microenvironment-derived cytokines reported to enhance CLL viability and drug resistance (37), could subvert the activity of EC-7072. As shown in Figure 3G, none of the soluble factors tested significantly affected the leukemia cell death brought about by the mithralog. Furthermore, co-culture of PBMCs from patients with CLL in the presence of OP9 stromal cells to mimic a protective microenvironmental niche did not enhance leukemia cell viability upon EC-7072 treatment (Figure 3H). Exposure of OP9 cells to EC-7072 for 24 h did not affect their viability (data not shown).

Altogether, these results indicate that the cytotoxic activity of EC-7072 against primary CLL cells entails caspase activation and that this antileukemic activity is not prevented by microenvironmental stimulatory signals.

### EC-7072 Modulates the Transcriptome of Primary CLL Cells

**Figure 4:**
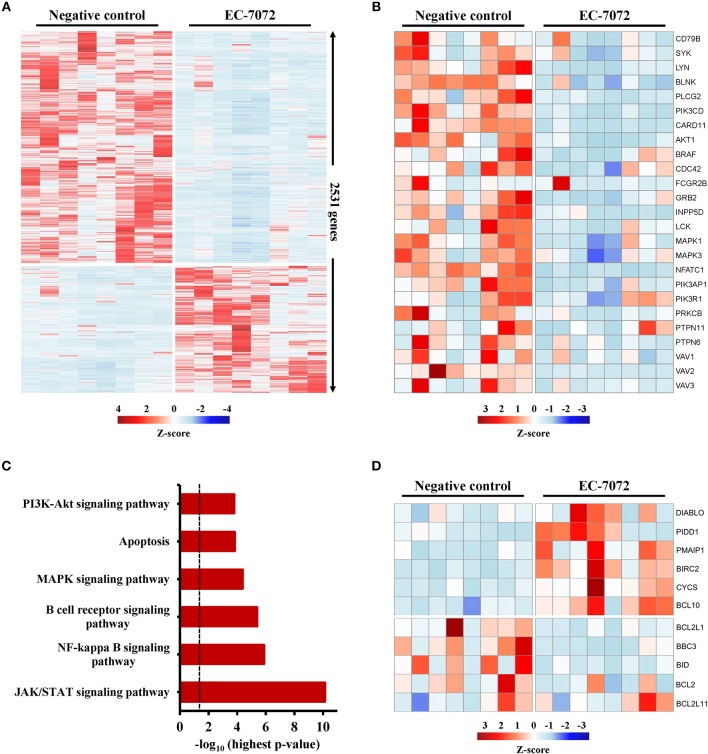
EC-7072 modulates the transcriptome of primary CLL cells. Isolated leukemia cells from patients with CLL (*n* = 8) were treated with 200 nM EC-7072 for 6 h. Total RNA was extracted and RNA-seq analysis was performed. **(A)** Heat map representation of statistically significant differences in gene expression between negative control (DMSO) and EC-7072-treated samples. The color scale represents the per-gene Z-score. Genes were selected based on adjusted *p*-value < 0.05, log_2_ (fold change) < −1 or > 1. **(B)** Heat map representation of expression of representative genes corresponding to BCR signaling that are significantly dysregulated by EC-7072 in CLL cells. The color scale represents the per-gene Z-score. **(C)** Selected relevant pathways for CLL cell homeostasis significantly modulated by EC-7072 obtained through KEGG pathway analysis. Bars represent the –log_10_ of the highest *p*-value obtained from the iterative pathway analysis (dashed line corresponds to *p*-value = 0.05). **(D)** Heat map representation of expression of genes related to apoptosis regulation that are significantly dysregulated by EC-7072 in CLL cells. The color scale represents the per-gene Z-score.

To elucidate the mechanisms underpinning the cytotoxic effect of EC-7072 against CLL cells, we interrogated the transcriptional profile of primary leukemia cells from 8 patients with CLL exposed to 200 nM EC-7072 for 6 h. RNA-seq analysis identified 2,531 differentially expressed genes in EC-7072-treated compared with control (DMSO) leukemia cells, hence demonstrating that the compound dramatically impacts the transcriptome of CLL cells (Figure 4A). Functional profiling of these transcripts revealed that EC-7072 modulates the expression of key mediators of signaling pathways that are paramount for CLL cell homeostasis and survival. Thus, exposure to EC-7072 resulted in a broad downregulation of genes required for functional BCR signaling, including members of the BCR complex (*CD79B*), BCR-proximal-related kinases (*SYK, LYN, PIK3CD*) and downstream signaling effectors (*PLCG2, CARD11*) (Figure 4B), which was subsequently validated by qPCR (Supplementary Figure 6A and Supplementary Table 5). Accordingly, KEGG pathway analysis of the protein-coding genes differentially expressed in CLL cells after EC-7072 treatment revealed an enrichment of multiple cascades engaged by the BCR-dependent signaling, such as NF-kB, JAK/STAT, PI3K/AKT, and MAPK pathways, with key roles in regulating gene transcription in CLL cells (38, 39) (Figure 4C, Supplementary Figure 6B and Supplementary Table 4). Dysregulation of these signaling networks entailed by impaired BCR-dependent signaling can lead to CLL cell demise by modulating the expression of molecules that govern apoptosis, a process that also scored among the most-enriched pathways in CLL cells upon treatment with EC-7072 (Figure 4D). Indeed, despite the mithralog did not trigger detectable cell death under these experimental settings (6 h, 200 nM EC-7072) (Supplementary Figure 6C), a marked shift in the expression profile of genes that govern apoptosis was already observed, thereby underscoring the ability of EC-7072 to subvert CLL cell viability (Figure 4D and Supplementary Figure 6D). In agreement, Western blotting analysis showed an increase in the levels of the proapoptotic protein Noxa, while the antiapoptotic protein BCL-xL was decreased, in primary leukemia cells exposed to EC-7072 in a time-dependent manner, further supporting the proapoptotic properties of the mithralog in CLL (Supplementary Figure 6E). Among these apoptotic regulators, it is worth mentioning that the gene expression of *BCL2*, an antiapoptotic protein overexpressed in CLL associated to leukemia cell survival and therapy resistance (40), was significantly downregulated in primary CLL cells after treatment with EC-7072 (Supplementary Figure 7A). Accordingly, a mild, but significant, decrease in the protein levels of BCL2 was observed on EC-7072-treated CLL cells by intracellular flow cytometry (Supplementary Figure 7B). Conversely, *BCL2* expression was not modulated by the compound in T cells isolated from patients with CLL, thereby reinforcing the notion that EC-7072 is highly efficient at inducing leukemia cell apoptosis compared to other immune cell subsets in patients with CLL (Supplementary Figures 7C,D).

Altogether, these results reveal that EC-7072 shapes the transcriptional landscape of primary CLL cells, reprogramming the expression of genes that control cell survival toward a pro-apoptotic state.

### EC-7072 Inhibits Tonic BCR Pathway by Suppressing the Expression and Phosphorylation of Key Signaling Nodes

**Figure 5:**
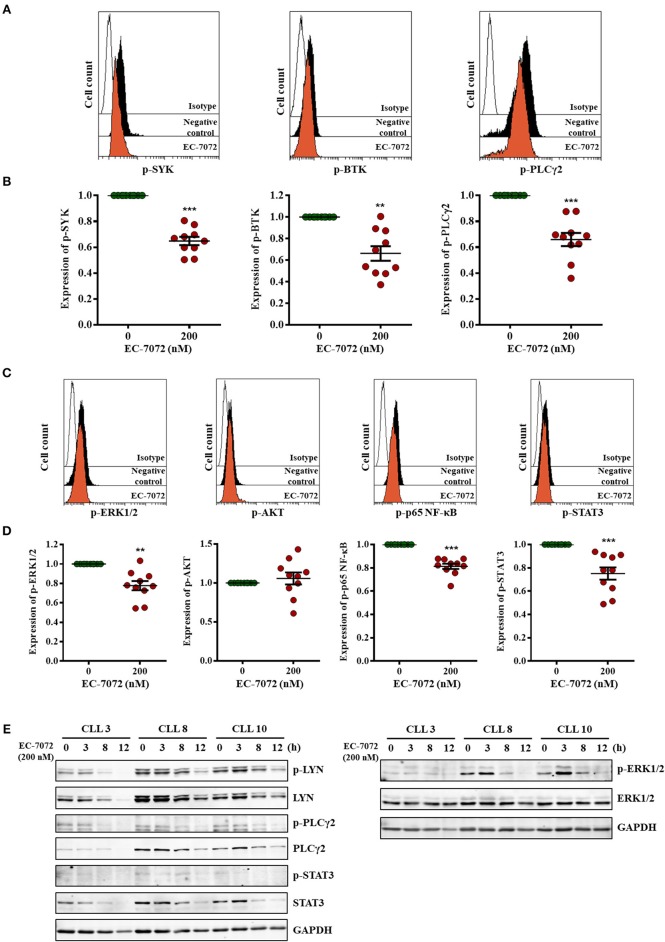
EC-7072 inhibits tonic BCR pathway by suppressing the phosphorylation of key signaling nodes. **(A–D)** Phosphorylation levels for the indicated proteins were evaluated by phosphoflow in isolated leukemia cells from patients with CLL (*n* = 10) incubated with EC-7072 (200 nM) for 8 h. Histograms depict the MFI from a representative patient **(A,C)**. Graphs represent the MFI normalized to the control (DMSO) from each individual patient and dark lines correspond to mean ± SEM **(B,D)** (^**^P < 0.01; ^***^P < 0.001; Student's *t*-test). **(E)** Isolated leukemia cells from 3 patients with CLL were treated with 200 nM EC-7072 for 3, 8 and 12 h. Protein lysates were immunoblotted to detect the indicated phosphorylated (p) and total protein levels.

Our RNA-seq analyses suggest that EC-7072 targets the BCR signaling axis by suppressing the expression of several components that operate at different levels of the cascade. Activation of the BCR signaling pathway is initiated by engagement of the BCR, a multimeric complex composed of a membrane immunoglobulin and the heterodimer CD79A/B (Igα/β) (36). In agreement with our transcriptomic studies we found that, despite the levels of CD79A remained unchanged after treatment, EC-7072 significantly decreased CD79B and IgM CLL cell surface expression (Supplementary Figure 8). Initial stimulation at the BCR complex leads to a phosphorylation cascade of intracellular mediators that amplifies the BCR signal (41). Therefore, upon demonstration that EC-7072 downregulates expression of BCR components, we sought to evaluate the phosphorylation of BCR signaling nodes by phosphospecific flow cytometry analysis. As previously reported, basal levels of phosphorylation of signaling mediators of the BCR cascade were highly heterogeneous in primary leukemia cells from patients with CLL (42). Nevertheless, as shown in Figures 5A,B, these experiments revealed a significant reduction of the levels of phosphorylated (p)-SYK, p-BTK and p-PLCγ2 in isolated leukemia cells after 8 h of incubation with EC-7072, a treatment that does not result in noticeable CLL cell death (Supplementary Figure 9A). The activity of these proximal signaling nodes is in turn required for stimulation of downstream pathways that sustain B-cell survival (13). Accordingly, a significant downregulation was registered in the phosphorylation levels of the effector kinase ERK1/2 and the transcription factors p65 NF-κB and STAT3 (Figures 5C,D) upon exposure to EC-7072, which is in line with the results of our RNA-seq analysis. No marked changes were detected in the amounts of p-AKT, suggesting that the effect of EC-7072 on leukemia cells is not likely to involve modulation of AKT phosphorylation (Figures 5C,D). As expected, treatment with recombinant CD40L or IL-6 strongly increased the basal phosphorylation levels of p65 NF-κB and STAT3, respectively, hence supporting that our phosphoflow analyses were reliable and robust (Supplementary Figure 9B). In agreement with these results, a time-dependent reduction of p-LYN, p-PLCγ2 and p-ERK1/2 was also evidenced by Western blotting (Figure 5E and Supplementary Figure 10A). Likewise, the levels of p-STAT3, albeit low, diminished upon exposure to EC-7072 (Figure 5E and Supplementary Figure 10A), which was further confirmed in the CLL-derived cell line MEC-1 (Supplementary Figure 9C). Concomitantly, EC-7072 was able to downregulate the total amount of LYN, PLCγ2 and STAT3 in CLL cells, further reinforcing the broad transcriptional reprogramming of primary leukemia cells from patients with CLL upon exposure to the compound (Figure 5E and Supplementary Figure 10A). Of note, except for ERK1/2, the decrease in the phosphorylation levels of these proteins clearly correlated with the observed reduction of their total amount, suggesting that EC-7072 might be mainly hindering BCR signaling via downregulation the expression of key signaling mediators of the pathway (Supplementary Figure 10B).

Altogether, these data suggest that EC-7072 impairs tonic BCR signaling in primary CLL cells by suppressing the expression and function of multiple key BCR pathway nodes.

### Activation of the BCR Partially Abrogates EC-7072-Induced Cell Death of Primary CLL Cells

**Figure 6:**
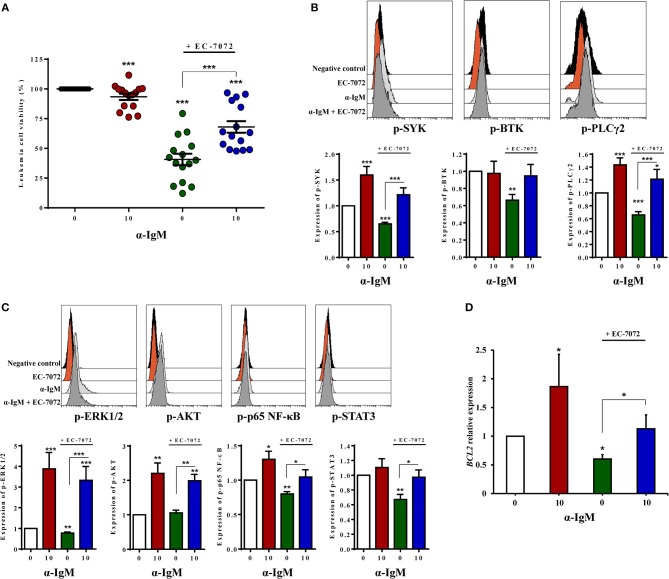
Activation of the BCR partially abrogates EC-7072-induced cell death of primary CLL cells. **(A)** Leukemia cell viability was determined by cytofluorometric assessment of DiOC_6_(3)/PI staining in PBMCs from patients with CLL (*n* = 15) incubated with EC-7072 (200 nM) in combination with α-IgM (10 μg/mL) for 24 h. The graph depicts the percentage of viable [DiOC_6_(3)^+^] cells normalized to their respective control (DMSO) condition for each individual patient and dark lines represent mean ± SEM. **(B)** Levels of phosphorylated SYK, BTK, and PLCγ2 were analyzed by phosphoflow in isolated leukemia cells from patients with CLL (*n* = 10) incubated with EC-7072 (200 nM) for 8 h followed by stimulation with α-IgM (10 μg/mL) for 15 min. Histograms depict the MFI from a representative patient and graphs show the MFI normalized to the control (DMSO) condition. **(C)** Levels of phosphorylated ERK1/2, AKT (*n* = 10), p65 NF-κB, and STAT3 (*n* = 6) were analyzed by phosphoflow in isolated leukemia cells from patients with CLL incubated with EC-7072 (200 nM) for 8 h followed by stimulation with α-IgM (10 μg/mL) for 15 min. Histograms depict the MFI from a representative patient and graphs show the MFI normalized to the control (DMSO) condition. **(D)** Isolated leukemia cells from patients with CLL (*n* = 4) were incubated with EC-7072 (200 nM) in combination with α-IgM (10 μg/mL) for 6 h and total RNA was extracted. Relative expression of *BCL2* was analyzed by qPCR. Bars represent the mRNA relative expression normalized to the control (DMSO) condition. (Mean ± SEM) (^*^P < 0.05; ^**^P < 0.01; ^***^P < 0.001; Student's *t*-test).

Based on the observation that EC-7072 subverts BCR signaling in CLL cells, we next asked whether this suppressive role underpins the antileukemic activity of the mithralog. We thus reasoned that activation of the BCR may counteract the cytotoxic effect of EC-7072 against primary CLL cells from patients. As shown in Figure 6A, BCR stimulation with anti-IgM antibodies significantly rescued leukemia cell survival in the presence of EC-7072. As expected, BCR crosslinking strongly induced the phosphorylation of downstream mediators of the pathway (Figures 6B,C). Accordingly, BCR stimulation resulted in increased levels of phosphorylated SYK and PLCγ2 in CLL cells treated with EC-7072 compared to unstimulated CLL cells exposed to the mithralog (Figure 6B). Furthermore, the enhanced phosphorylation of ERK1/2, AKT, p65 NF-κB, and STAT3 ensued by treatment with anti-IgM was not significantly reverted by EC-7072 (Figure 6C). Noteworthy, we found that BCR activation was able to restore the expression of *BCL2* to basal levels in isolated CLL cells treated with the mithralog, further supporting the pro-survival role of anti-IgM by antagonizing the pro-apoptotic activity of EC-7072 against leukemia cells (Figure 6D).

Overall, these experiments show that stimulation of the BCR, as illustrated by the enhanced phosphorylation of BCR signaling downstream mediators detected, counteracts the cytotoxicity of EC-7072 against primary CLL cells, hence supporting that the mithralog negatively modulates tonic BCR signaling.

### EC-7072 Displays a Cytotoxic Activity Against Primary CLL Cells Analogous and Additive With That of Therapies Routinely Used in CLL

**Figure 7:**
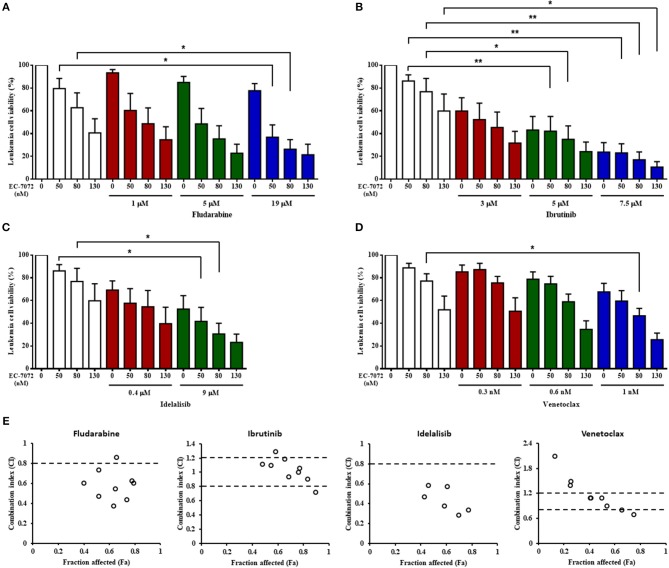
The cytotoxicity of EC-7072 against CLL cells is comparable to therapies routinely used in CLL. **(A–D)** Leukemia cell viability (*n* = 6) was assessed by DiOC_6_(3)/PI staining upon exposure to non-fixed ratio combinations of EC-7072 with fludarabine **(A)**, ibrutinib **(B)**, idelalisib **(C)**, or venetoclax **(D)**. Concentrations employed correspond to IC_10_, IC_25_, and IC_50_ values calculated for each compound. Bars represent the percentage of viable [DiOC_6_(3)^+^] cells normalized to their respective control (DMSO) condition (mean ± SEM) (^*^P < 0.05; ^**^P < 0.01; One-way ANOVA). **(E)** CI plots generated for non-fixed ratio combinations of EC-7072 and fludarabine, ibrutinib, idelalisib, or venetoclax using CalcuSyn software according to the Chou-Talalay method. CI values <0.8 indicate synergism, values between 0.8 and 1.2 correspond to additive effects and values >1.2 represent antagonism. The fraction affected (Fa) was calculated based on the percentage of viable cells after the treatment.

Our assays show that EC-7072 downregulates the expression and activation of several components of the BCR signaling pathway that are targets of recent therapeutic approaches for CLL. Thus, we evaluated whether the *ex vivo* antileukemic efficacy of EC-7072 is comparable to that of therapies currently approved for patients with CLL. PBMCs from patients with CLL were treated with EC-7072, fludarabine or the targeted agents ibrutinib (15), idelalisib (16) or venetoclax (40) in a range of previously-employed doses (15, 16, 43) and leukemia cell viability was assessed by DiOC_6_(3)/PI staining and flow cytometric detection. We found that treatment with EC-7072 exerted similar cytotoxicity against primary CLL cells than that of ibrutinib or venetoclax, but reduced CLL cell viability to a greater extent than idelalisib or fludarabine under the experimental settings tested (Supplementary Figure 11A). To determine the combined efficacy, PBMCs from patients with CLL were incubated with non-fixed ratio combinations of these compounds, employing IC_10_, IC_25_, and IC_50_ doses. EC-7072 enhanced the effect of the four therapeutic agents tested in killing primary CLL cells (Figures 7A–D) and calculation of CI values unraveled a general additive effect of the mithralog with ibrutinib and venetoclax, whereas combination with idelalisib or fludarabine exerts synergistic cytotoxicity on leukemia cells (Figure 7E). The synergistic effect observed upon combination with idelalisib suggests that both compounds might be targeting distinct signaling pathways, which would correlate with our previous data supporting that EC-7072 is not likely inhibiting AKT signaling.

Additionally, the impact of the combined treatments, used at previously-described concentrations (15, 16, 43), on the survival of healthy immune subsets, T cells and NK cells, was evaluated. EC-7072 was significantly less toxic than fludarabine and ibrutinib against T cells and NK cells from patient's PBMCs (Supplementary Figures 11B,C). Idelalisib and venetoclax exerted negligible effects on the viability of these populations, similarly to EC-7072 (Supplementary Figures 11D,E). Of note, the combination treatment resulted in incremented toxicity against NK cells (Supplementary Figures 11B–E).

Collectively, our assays indicate that EC-7072 displays analogous or higher *ex vivo* antileukemic activity than that of drugs currently employed for CLL therapy and shows an additive or synergistic effect with these agents, suggesting that the use of this mithralog alone or in combination regimens may represent a novel therapeutic approach for CLL.

## Discussion

Herein, we provide evidence that the MTA analog EC-7072 exerts a potent antileukemic *in vitro* activity in CLL at nanomolar concentrations. In line with previous *in vivo* studies demonstrating that EC-7072 is well-tolerated in mice compared to MTA (25, 26), we found that the mithralog is substantially less toxic than the parental compound against human healthy cells of different tissue origins. Conversely, EC-7072 induced death of both non-malignant and leukemia B cells, underscoring a selectivity of the compound to target this immune cell subset. Indeed, treatment of PBMCs from patients with CLL revealed significantly higher cytotoxicity of EC-7072 against leukemia cells compared to circulating NK cells and, mainly, T cells. Furthermore, the mithralog did not alter the immune production of key antitumor effector molecules (IFN-γ and perforin), suggesting that EC-7072 does not exacerbate the immune dysregulation frequently observed in patients with CLL. Nevertheless, similar cell death was observed when isolated CLL cells were exposed to EC-7072 in the absence of other immune cell subsets, indicating that the mithralog directly elicits leukemia cell demise.

**Figure 8:**
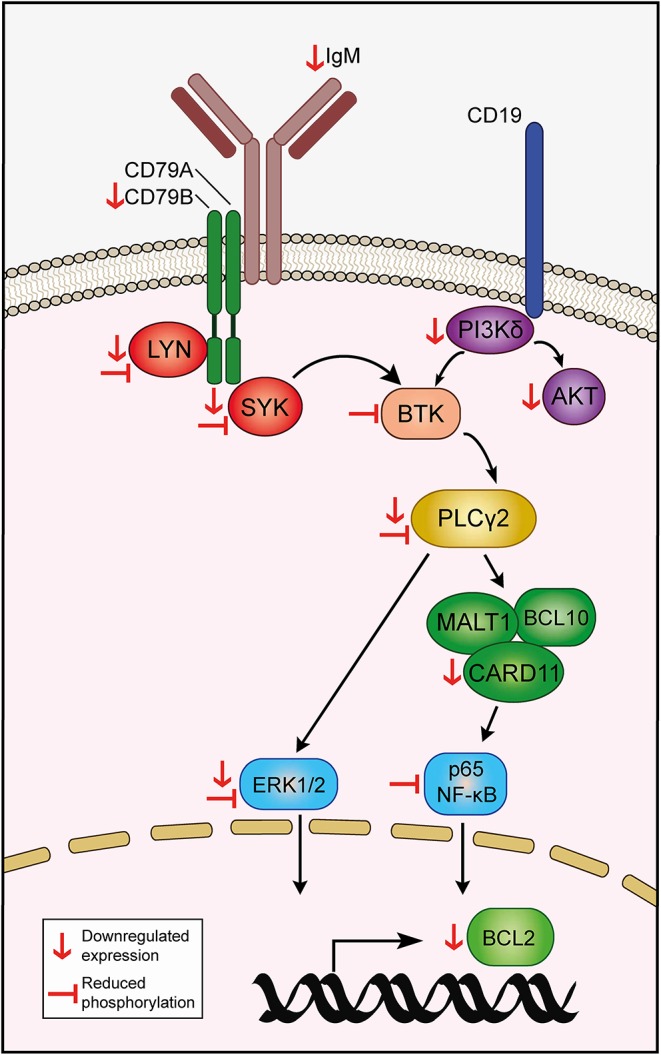
Hypothetical model of action of EC-7072 targeting the BCR signaling pathway in CLL cells. Red downwards arrows (↓) represent downregulation of protein or transcriptional expression in CLL cells upon EC-7072 treatment. Left tack symbols (**⊣**) represent reduced phosphorylation levels of key components of the BCR cascade in primary CLL cells after exposure to EC-7072.

Leukemia and non-malignant B cells are heavily dependent on the BCR signaling pathway for survival. Signaling through the BCR propagates via phosphorylation of downstream mediators, eventually resulting in constitutive activation of signaling pathways -such as NF-κB, JAK/STAT, and MAPK- that sustain leukemia cell viability by, at least in part, upregulating the expression of antiapoptotic members of the BCL2 family of proteins (44). Consequently, disruption of the BCR pathway by blocking the mediators of the cascade often results in CLL cell death, which is the mainstay of the therapeutic efficacy of targeted agents employed in the treatment of CLL (12). Our RNA-seq studies revealed that EC-7072 may target the BCR signaling pathway at multiple levels in primary CLL cells from patients (Figure 8). Thus, exposure to the mithralog resulted in a broad downregulation of protein-coding genes of components of the BCR complex (*CD79B*), BCR-proximal kinases (*SYK, LYN, PIK3CD*) and downstream signaling effectors (*PLCG2, CARD11*). Accordingly, signaling pathways that are engaged downstream of the BCR cascade (including NF-κB, JAK/STAT, and MAPK) were found altered in KEGG analysis of the differentially expressed genes, suggesting a profound dysregulation of the BCR signaling pathway in CLL cells brought about by EC-7072. The mechanism underpinning the EC-7072-driven reprogramming of the transcriptome of primary CLL cell merits future investigation. Consistent with our experiments demonstrating that EC-7072 elicits apoptotic CLL cell death in a caspase-3-dependent manner, the mithralog reprogrammed leukemia cell expression of a set of genes that control apoptosis, including the downregulation of BCL2 gene and protein expression, an antiapoptotic protein highly expressed in CLL cells that is an attractive target for CLL therapies (45), and a pronounced increase in Noxa levels, a proapoptotic protein commonly upregulated in CLL as a result of treatment with apoptosis-inducing agents (46–48). Overstimulation of the BCR signaling by antibody-mediated receptor crosslinking was able to largely counteract the antileukemic activity of EC-7072. A hallmark of several B-cell malignancies is the chronic activation of this pathway observed in patients, which might represent a hindrance for the therapeutic application of this mithralog. However, our study demonstrates that exposure to microenvironment-derived factors, as well as co-culture with stromal cells, which are known to mediate therapy resistance in patients with CLL, failed to counteract the antileukemic activity of EC-7072, hence suggesting that the efficacy of the compound might not diminish *in vivo*. Nonetheless, the behavior and the antileukemic activity of EC-7072 *in vivo* remain to be fully elucidated.

The functional relevance of the impact of the mithralog on the transcriptional landscape of primary CLL cells was further evidenced by demonstration of reduced levels of phosphorylated signaling nodes and pathways downstream of the BCR, suggesting that EC-7072 may hamper constitutive tonic activation of BCR-dependent signaling cascades. Such an effect is in line with our finding that EC-7072 rapidly reduces the surface expression of BCR subunits (CD79B and IgM), although the precise role of these molecules in the activity of the compound in CLL cells remains to be determined. Nevertheless, we found that BCR stimulation is able to antagonize the repression of downstream signaling nodes and, noteworthy, significantly counteract the apoptotic cell death triggered by EC-7072, which was accompanied by upregulation of *BCL2* gene expression, further supporting that the antileukemic activity of the compound may involve modulation of the basal BCR signaling status and the proapoptotic/antiapoptotic balance in CLL cells.

Most of the novel therapies for treatment of CLL are targeted agents that block mediators of the BCR signaling pathway, several being also downregulated by EC-7072. However, a major pitfall of CLL therapies is the development of therapeutic resistance in high-risk patients, which prompted the design of combination therapies (18, 19). Of relevance, CLL cell death induced by EC-7072 was not affected by the presence of molecular and cytogenetic aberrations found in patients with CLL refractory to certain chemotherapeutic strategies or the use of soluble microenvironment-derived factors that sustain leukemia cell survival (49). Furthermore, acquired resistance to targeted CLL therapies is frequently conferred by mutations in genes encoding BCR signaling mediators. Thus, co-occurring mutations in *BTK* and *PLCG2* have been shown to mediate ibrutinib resistance (50), while a point mutation in *BCL2* has recently been identified in patients with CLL resistant to treatment with venetoclax (19). The dramatic impact of EC-7072 on the transcriptome of CLL cells opens the question of whether the mithralog would be effective in the presence of mutational events affecting components of the BCR signaling pathway that are targets of these novel agents, hence supporting its use in combined CLL therapies. Indeed, we found that EC-7072 displays comparable or higher killing activity on CLL cells than that of fludarabine, ibrutinib, idelalisib and venetoclax, used at previously-reported doses (15, 16, 43). Moreover, an additive or synergistic activity of EC-7072 with the compounds tested was observed. Conversely, the mithralog was significantly less toxic to non-malignant immune cells from the same patients than other agents, mainly fludarabine, which frequently causes lymphocytopenia in patients with CLL (10). Given the interesting combined efficacy obtained upon co-treatment with idelalisib, elucidating the precise role of PI3K/AKT signaling on the antileukemic effect of EC-7072 would be of great interest in future studies. In this sense, and given that no changes in the phosphorylation levels of AKT1 at S473 were observed, the effect of the compound on additional residues known to be phosphorylated in response to AKT1 activation (e.g., T308) might bring to light a novel aspect of the antileukemic activity of EC-7072 in CLL.

Overall, our findings provide evidence that the mithralog EC-7072 induces leukemia cell death by hampering the proficiency of BCR signaling in CLL cells. The compound enhances the antileukemic activity of approved therapeutic agents and is effective independent of the IGHV mutational status or interphase cytogenetics, hence opening the question of whether EC-7072 may be a potential novel standalone or combination therapeutic option for patients with CLL and other B-cell malignancies.

## Data Availability Statement

The datasets generated for this study can be found in Gene Expression Omnibus database, GSE123777.

## Ethics Statement

Written informed consent was obtained from all the patients following the Declaration of Helsinki and samples were collected with approval from the local ethics committee (Comité de Ética de la Investigación del Principado de Asturias, case-19042016).

## Author Contributions

SL-H designed and performed all the experiments, analyzed data, and wrote the manuscript. CS-B performed experiments and analyzed data. GB performed Western blot experiments and analyzed data. ÁP, AG-R, and EG-G provided samples and clinical data. JP-E analyzed data. L-EN and FM generated and provided reagents necessary for this study. SG designed experiments and wrote the manuscript. AL-S conceptualized the study, designed experiments, supervised the research, and wrote the manuscript. All the authors reviewed and approved the final version of the manuscript.

### Conflict of Interest

FM has a leadership role and holds ownership interest in EntreChem S.L. L-EN, CS-B, and JP-E receive salary from EntreChem S.L. SL-H, CS-B, JP-E, L-EN, FM, SG, and AL-S are inventors in a patent application for the use of EC-7072 and synergistic compositions thereof in the treatment of chronic lymphocytic leukemia. The remaining authors declare that the research was conducted in the absence of any commercial or financial relationships that could be construed as a potential conflict of interest.
