# Supplementary material for: The Mithralog EC-7072 Induces Chronic Lymphocytic Leukemia Cell Death by Targeting Tonic B-Cell Receptor Signaling
Source: Front Immunol. 2019 Oct 18;10:2455. doi: 10.3389/fimmu.2019.02455 (PMC6813538; doi:10.3389/fimmu.2019.02455)
Supplement: Supplementary file 1 [file Data_Sheet_1.docx]

***Supplementary material***

**1. Supplementary Methods**

**Reagents and cell culture**

Mithramycin A (MTA) was provided by EntreChem S.L. (Oviedo, Spain). Ibrutinib, idelalisib, venetoclax and fludarabine were obtained from Selleckchem. Recombinant human (rh) TNF-α, IL-6, IL-4 and BAFF were purchased from Peprotech. Soluble multimeric CD40L was obtained from Adipogen.

All cell lines were obtained from ATCC and cultured in the recommended culture medium supplemented with 10% heat-inactivated FBS (Sigma-Aldrich), 1 mM sodium pyruvate, 2 mM L-glutamine, 100 U/mL penicillin and 10 µg/mL streptomycin at 37^o^C and 5% CO_2_. HK-2 cells were further supplemented with 36 ng/mL hydrocortisone and Insulin-Transferrin-Selenium (ITS; Gibco). Healthy fibroblasts were obtained from healthy donors and kindly provided by Dr. Isabel Quirós (Universidad de Oviedo, Spain).

**Intracellular staining**

PBMCs from patients with CLL were treated with 200 nM EC-7072 or DMSO for 24 hours and stimulated with PMA (50 ng/mL) and ionomycin (1 µg/mL) for 5 hours in the presence of GolgiPlug protein transport inhibitor (Beckton Dickinson). T cells and NK cells were then analyzed for intracellular levels of IFN-γ and perforin by flow cytometry.

For BCL2 staining, PBMCs from patients with CLL were exposed to 200 nM EC-7072 for 12 and 18 hours. After immune subset staining, cells were fixed, permeabilized and stained with anti-human BCL2-PE (clone: BCL/10C4; Biolegend). Results were analyzed by flow cytometry.

**qPCR analysis**

Isolated leukemia cells from patients with CLL were incubated with EC-7072 (200 nM) or DMSO for 6 hours. Total RNA was extracted using RNeasy Mini Kit (Qiagen). 2 µg total RNA were used for cDNA synthesis using High-Capacity cDNA Reverse Transcription Kit (Applied Biosystems). Quantitative RT-PCR was performed using SYBR Green PCR Master Mix (Applied Biosystems) in a 7300 Real-Time PCR System and each sample was analyzed in triplicate. Primers used for each gene detection are provided in **Supplementary Table 5**.

**Western blotting quantification**

Levels of phosphorylated and total proteins were quantified by densitometric analysis employing ImageJ software (https://imagej.nih.gov/ij/). To determine relative protein amounts, signal intensities were first normalized to GAPDH and the ratio between phosphorylated and total proteins was then calculated.

**2. Supplementary Tables**

**Supplementary Table 1. Clinical characteristics of patients with CLL**

| **Study label** | **Gender (F, female; M, male)** | **Age (years) (Mean age = 72)** | ***IGHV* status** | **Cytogenetic alterations** | **RAI** | **Binet** |
| --- | --- | --- | --- | --- | --- | --- |
| CLL 1 | M | 65 | M | del(13q) | I | A |
| CLL 2 | M | 78 | M | Normal | II | A |
| CLL 3 | F | 71 | M | Normal | 0 | A |
| CLL 4 | F | 69 | M | del(13q) | I | B |
| CLL 5 | F | 66 | M | Normal | I | B |
| CLL 6 | M | 81 | M | Normal | I | A |
| CLL 7 | M | 80 | M | del(13q) | 0 | A |
| CLL 8 | M | 52 | M | del(13q) | 0 | A |
| CLL 9 | M | 61 | M | Normal | I | A |
| CLL 10 | M | 67 | M | Normal | 0 | A |
| CLL 11 | M | 72 | U | del(17p) | II | B |
| CLL 12 | M | 84 | U | del(13q); del(17p) | IV | C |
| CLL 13 | M | 77 | M | Normal | III | B |
| CLL 14 | M | 78 | M | Normal | 0 | A |
| CLL 15 | F | 61 | M | del(13q) | 0 | A |
| CLL 16 | F | 72 | M | del(13q) | II | B |
| CLL 17 | F | 80 | M | del(13q) | n.d. | n.d. |
| CLL 18 | M | 59 | M | del(13q) | 0 | A |
| CLL 19 | M | 82 | M | del(17p); del(11q); trisomy 12 | IV | A |
| CLL 20 | M | 69 | U | del(11q) | IV | C |
| CLL 21 | F | 68 | M | del(13q) | III | C |
| CLL 22 | F | 64 | M | del(13q) | 0 | A |
| CLL 23 | F | 86 | M | n.d. | 0 | A |
| CLL 24 | M | 63 | M | Normal | I | A |
| CLL 25 | F | 76 | M | Normal | I | A |
| CLL 26 | M | 84 | M | del(13q) | 0 | A |
| CLL 27 | M | 84 | U | del(13q) | I | B |
| CLL 28 | F | 70 | U | del(13q); del(17p) | 0 | A |
| CLL 29 | M | 75 | U | del(17p) | II | B |
| CLL 30 | F | 69 | M | del(13q); del(17p) | 0 | A |
| CLL 31 | M | 86 | M | Normal | IV | C |
| CLL 32 | M | 63 | M | trisomy 12 | 0 | A |
| CLL 33 | F | 88 | M | Normal | I | A |
| CLL 34 | M | 74 | M | del(13q) | I | A |
| CLL 35 | M | 71 | M | del(13q) | IV | C |
| CLL 36 | F | 70 | M | Normal | 0 | A |
| CLL 37 | F | 79 | M | Normal | II | A |
| CLL 38 | M | 69 | M | Normal | 0 | A |
| CLL 39 | M | 70 | M | Normal | I | A |
| CLL 40 | F | 83 | U | del(11q) | II | B |
| CLL 41 | M | 80 | U | del(13q); del(17p) | IV | C |
| CLL 42 | M | 68 | U | del(17p) | III | B |
| CLL 43 | M | 91 | M | trisomy 12 | IV | C |
| CLL 44 | M | 82 | M | Normal | 0 | A |
| CLL 45 | M | 71 | M | del(13q) | 0 | A |
| CLL 46 | M | 61 | U | Normal | I | A |
| CLL 47 | F | 71 | M | Normal | I | A |
| CLL 48 | M | 63 | M | Normal | 0 | A |
| CLL 49 | M | 64 | U | Normal | I | B |
| CLL 50 | F | 58 | U | del(13q); del(11q) | I | A |
| CLL 51 | M | 71 | M | Normal | 0 | A |
| CLL 52 | M | 66 | M | del(13q); trisomy 12 | I | A |
| CLL 53 | M | 77 | U | Normal | IV | A |
| CLL 54 | M | 60 | M | del(13q) | 0 | A |
| CLL 55 | M | 63 | U | del(11q); trisomy 12 | II | B |
| CLL 56 | F | 74 | U | del(13q) | II | B |
| CLL 57 | M | 85 | M | Normal | IV | B |
| CLL 58 | M | 63 | U | n.d. | IV | C |
| CLL 59 | F | 74 | n.d. | Normal | 0 | A |
| CLL 60 | F | 77 | n.d. | del(13q) | II | B |
| CLL 61 | F | 75 | n.d. | Normal | 0 | A |
| CLL 62 | F | 53 | M | Normal | 0 | A |
| CLL 63 | F | 81 | n.d. | del(13q) | 0 | A |

n.d. data unavailable.

**Supplementary Table 2. Antibodies used for phosphoflow**

| **Antibody** | **Fluorophore** | **Catalog** | **Residue** | **Clone** | **Manufacturer** |
| --- | --- | --- | --- | --- | --- |
| p-SYK* | PE | 683703 | Y352 | 1503310 | Biolegend |
| p-BTK | PE | 601703 | Y223 | A16128B | Biolegend |
| p-PLCγ2 | PE | 558490 | Y759 | K86-689.37 | BD Bioscience |
| p-ERK1/2 | PE | 369505 | T202/Y204 | 6B8B69 | Biolegend |
| p-AKT | PE | 561671 | S473 | M89-61 | BD Bioscience |
| p-p65 NF-κB | PE | 558423 | S529 | K10-895.12.50 | BD Bioscience |
| p-STAT3 | PE | 612569 | Y705 | 4/P-STAT3 | BD Bioscience |
| p-H2AX | PE | 613411 | S139 | 2F3 | Biolegend |

*This antibody also detects phosphorylated ZAP70 at Y319. As previously reported, phosphorylation of ZAP70 is highly inefficient in CLL and, therefore, signal detected by this antibody can be mostly attributed to SYK phosphorylation at Y352 ([1](#_ENREF_1), [2](#_ENREF_2)).

**Supplementary Table 3. Antibodies employed for western blotting**

| **Antibody** | **Catalog** | **Clone** | **Manufacturer** |
| --- | --- | --- | --- |
| p-PLCγ2 (Y759) | 3874 | n.a. | Cell Signaling |
| p-ERK1/2 (T202/Y204) | 4377 | 197G2 | Cell Signaling |
| p-STAT3 (Y705) | 9145 | D3A7 | Cell Signaling |
| p-LYN (Y507) | 2731 | n.a. | Cell Signaling |
| PLCγ2 | sc-52803 | B-10 | Santa Cruz Biotechnology |
| ERK1/2 | sc-514302 | C-9 | Santa Cruz Biotechnology |
| STAT3 | 9139 | 124H6 | Cell Signaling |
| LYN | sc-7274 | H-6 | Santa Cruz Biotechnology |
| BCL-xL | sc-56021 | 7B2.5 | Santa Cruz Biotechnology |
| Noxa | sc-56169 | 114C307 | Santa Cruz Biotechnology |
| GAPDH | 2118 | 14C10 | Cell Signaling |

n.a. not applicable. These antibodies are polyclonal.

**Supplementary Table 5. Primers used for qPCR analyses**

| **Gene** | **Forward sequence** | **Reverse sequence** |
| --- | --- | --- |
| *CD79B* | CCAGGCTGGCGTTGTCTCCTG | GGTACCGGTCCTCCGATCTGGC |
| *SYK* | TGCACTATCGCATCGACAAAG | CATTTCCCTGTGTGCCGATTT |
| *LYN* | ACCAGGGAGGAGCCCATTTA | CTTCCGCTCGATGTATGCCA |
| *PIK3CD* | ACTCTGCCATTGTCTAAGCCACCT | TCACAGCAGGTTCCCAAAGGTGAT |
| *PLCG2* | GCCTGTCCCTTTGTAGAAGTGG | GGCCATTATCATTCACAACCG |
| *BCL2* | CTGCACCTGACGCCCTTCACC | CACATGACCCCACCGAACTCAAAGA |
| *BCL2L11* | GGCCCCTACCTCCCTACA | GGGGTTTGTGTTGATTTGTCA |
| *BID* | CGCACCTACGTGAGGAGCTTAGCC | TGACCACATCGAGCTTTAGCCAGTCA |
| *BCL2L1* | GATCCCCATGGCAGCAGTAAAGCAAG | CCCCATCCCGGAAGAGTTCATTCACT |
| *PMAIP1* | CAGAGCTGGAAGTCGAGTGT | AGGAGTCCCCTCATGCAAGT |
| *CARD11* | TTGTGGGAGAATGTGGAGTGT | TGCCCCTTGGTATGTAGAATG |
| *B2M* | TGCTGTCTCCATGTTTGATGTATCT | TCTCTGCTCCCCACCTCTAAGT |

**3. Supplementary Figures**

**Supplementary Figure 1**

**
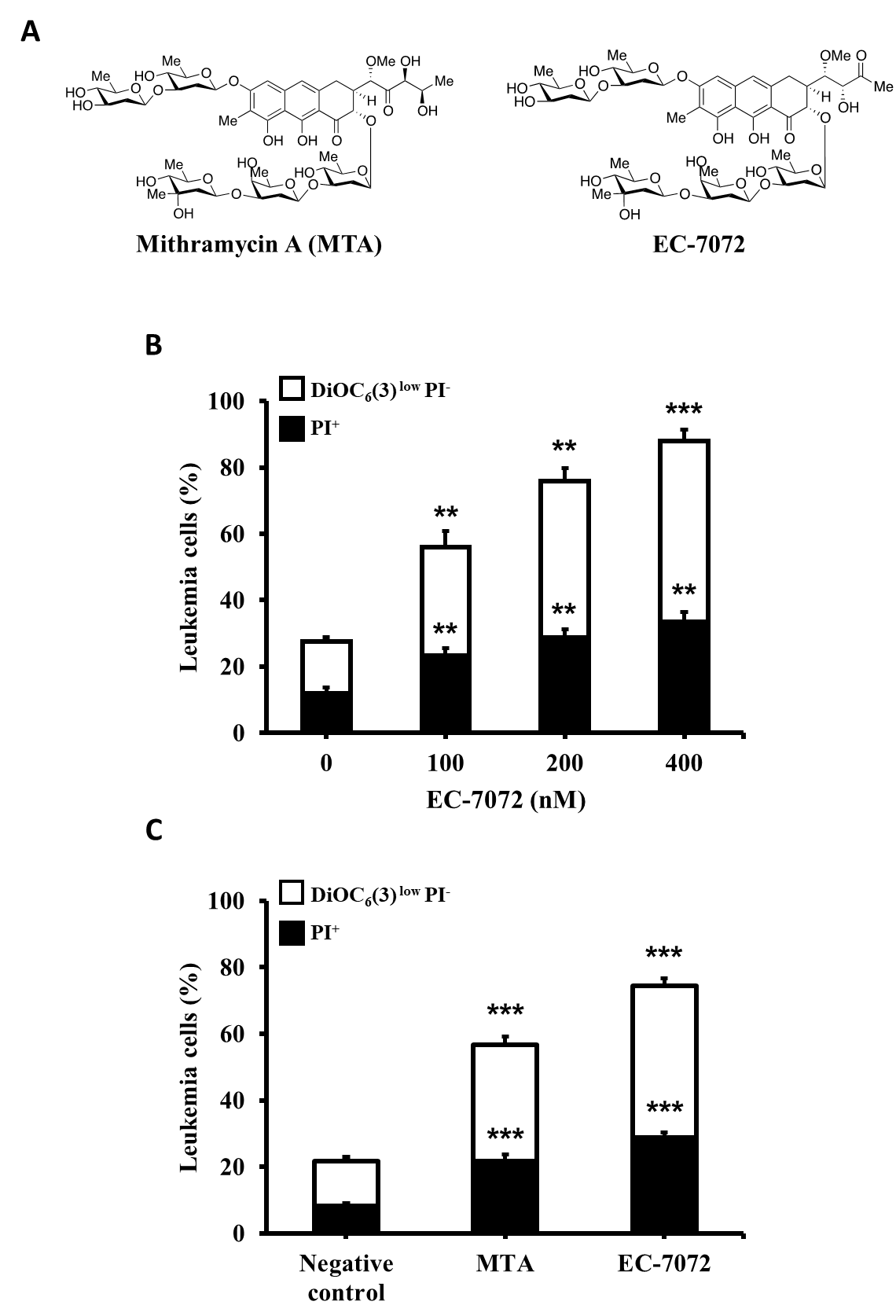
**

**Supplementary Figure 1.** **EC-7072 has a cytotoxic effect against primary CLL cells. (A)** Structure of mithramycin A (MTA) and its analog, EC-7072. **(B)** PBMCs from patients with CLL (n = 10) were incubated with increasing doses (0-400 nM) of EC-7072 for 24 hours. Apoptosis of leukemia cells was determined by DiOC_6_(3)/PI staining and flow cytometry. Bars represent the mean percentages of apoptotic [DiOC_6_(3)^low^ PI^-^] and dead [PI^+^] cells. **(C)** PBMCs from patients with CLL (n = 24) were treated with MTA (200 nM) or EC-7072 (200 nM) for 24 hours and leukemia cell death was evaluated by DiOC_6_(3)/PI staining. Bars represent the mean percentages of apoptotic [DiOC_6_(3)^low^ PI^-^] and dead [PI^+^] cells. (Mean ± SEM) (***P* < 0.01; ****P* < 0.001; Student’s *t*-test).

**Supplementary Figure 2**

**
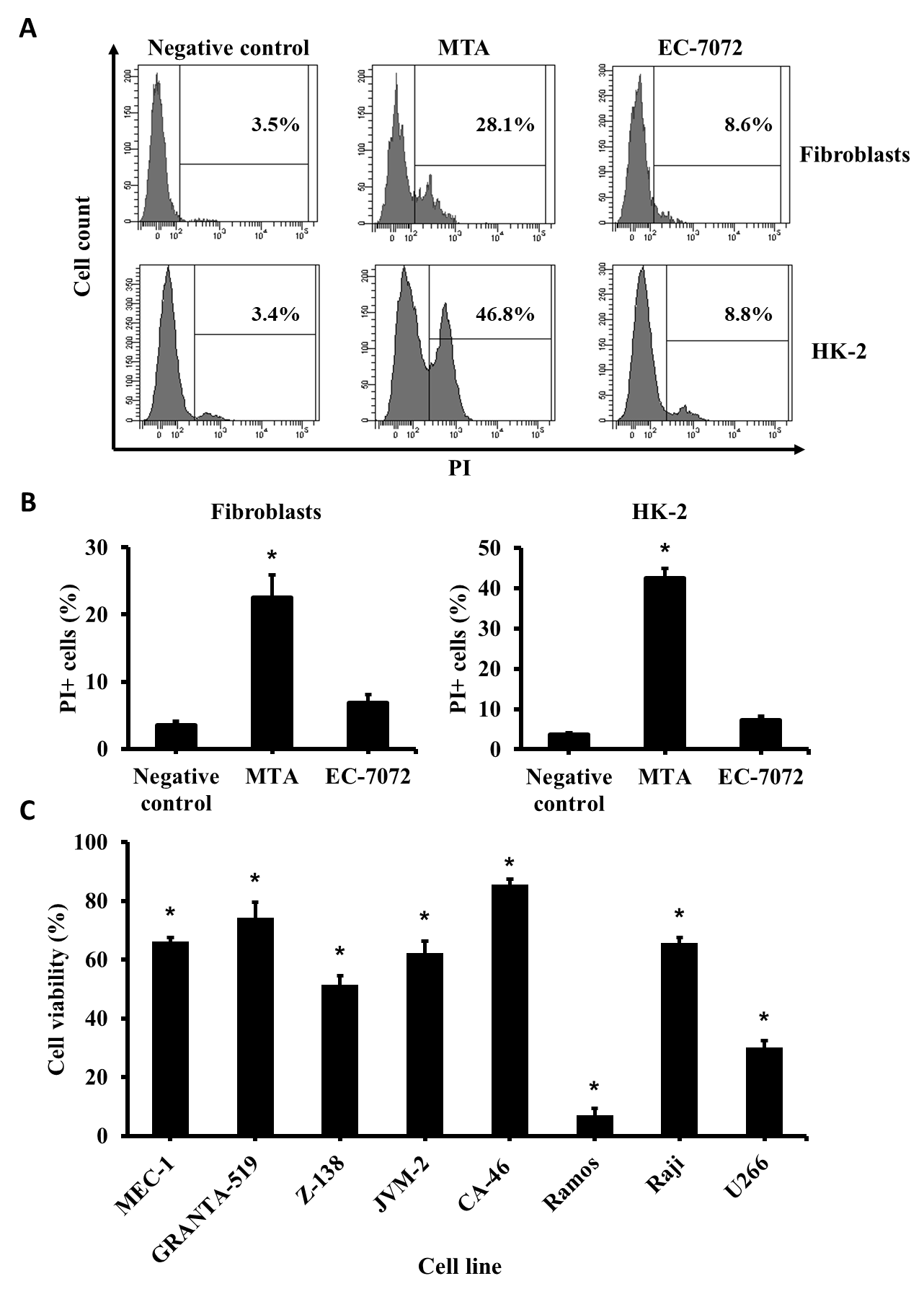
**

**Supplementary Figure 2. EC-7072 does not markedly affect non-tumoral cells but is effective against different B-cell malignancies. (A and B)** Primary fibroblasts from healthy donors (n = 4) and HK-2 cells from healthy human adult kidney (n = 3) were treated with MTA (200 nM) or EC-7072 (200 nM) for 24 hours and cell death was evaluated by PI staining. Histograms depict a representative experiment and percentages within correspond to the PI^+^ subset **(A)**. Bars represent the mean percentage of PI^+^ cells **(B)**. **(C)** Indicated cell lines were treated with EC-7072 (500 nM) for 24 hours and cell viability was assessed by DiOC_6_(3)/PI staining (n = 3). Bars represent the mean percentage of viable [DiOC_6_(3)^+^] cells normalized to control (DMSO) condition. (Mean ± SEM) (**P* < 0.05; Student’s *t*-test).

**Supplementary Figure 3**

**
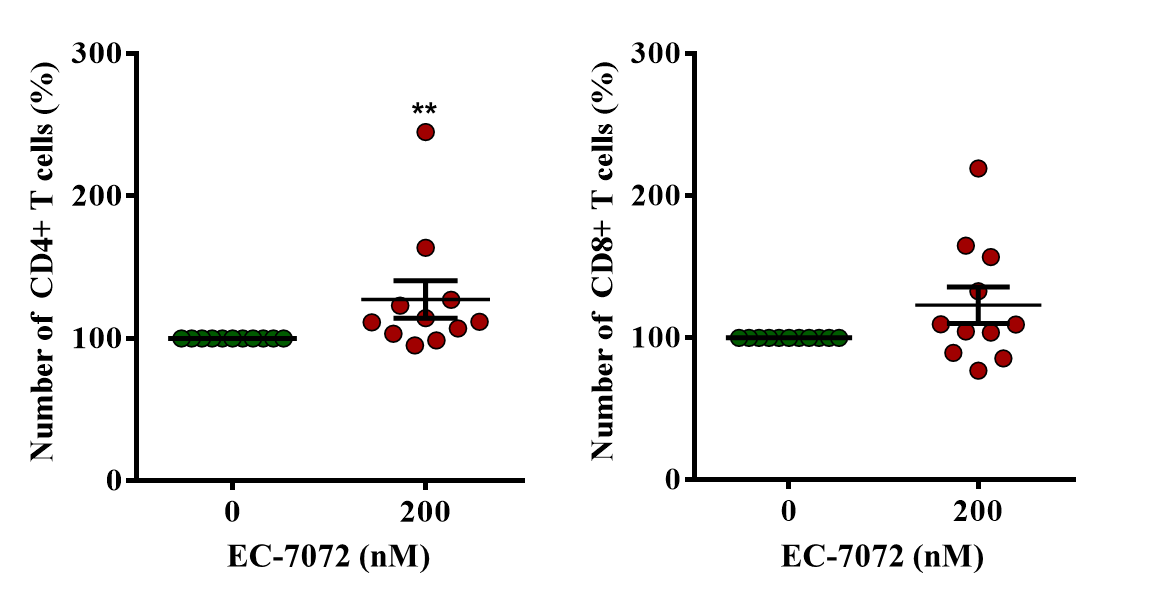
**

**Supplementary Figure 3. Effect of EC-7072 on the numbers of T lymphocytes from patients with CLL.** Numbers of CD4^+^ and CD8^+^ T cells were evaluated by flow cytometry in PBMCs from patients with CLL (n = 11) incubated with DMSO or EC-7072 (200 nM) for 24 hours. Graphs represent the number of cells normalized to their respective control (DMSO) condition for each individual experiment. Dark lines correspond to mean ± SEM (***P* < 0.01; Student’s *t*-test).

**Supplementary Figure 4**

**
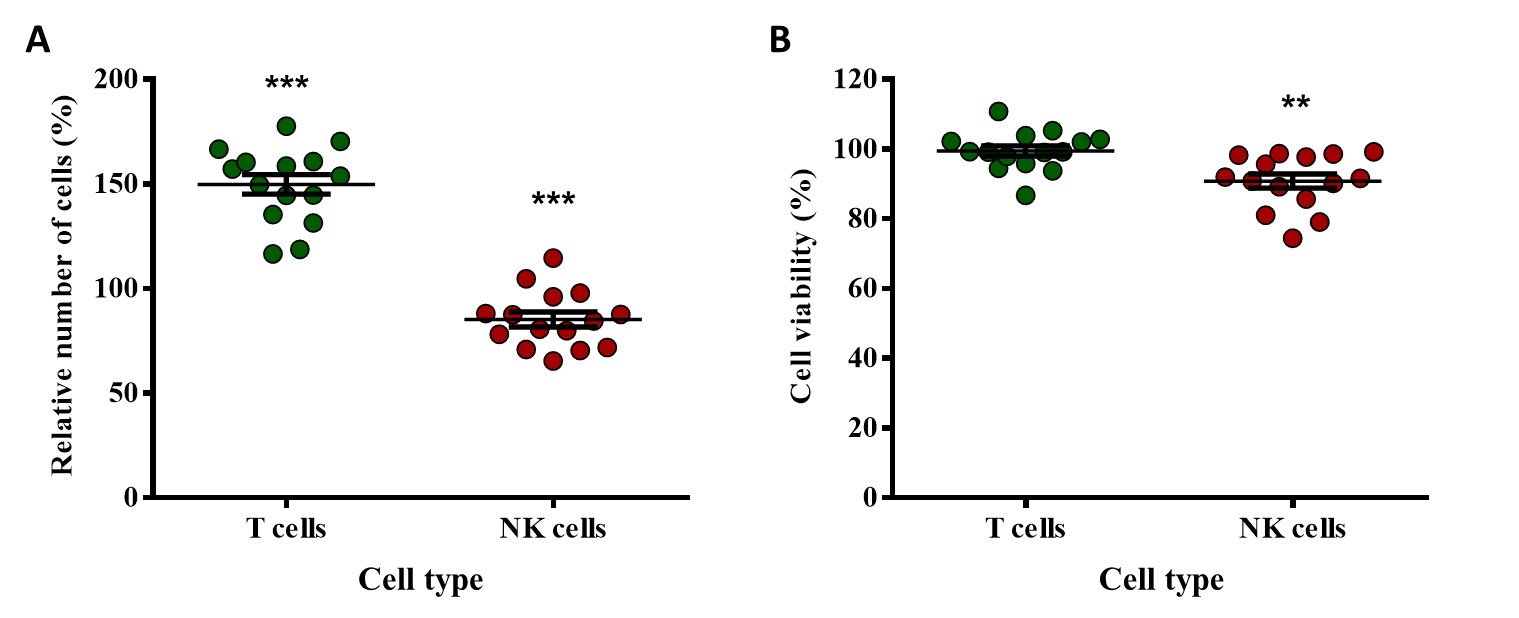
**

**Supplementary Figure 4. EC-7072 shows reduced toxicity on T lymphocytes and NK cells from healthy donors.** PBMCs from healthy donors (n = 15) were incubated with EC-7072 (200 nM) for 24 hours. **(A)** Numbers of T cells and NK cells were evaluated by flow cytometry. The graph represents the number of cells normalized to their respective control (DMSO) condition for each experiment. **(B)** Viability of immune subsets (T cells and NK cells) was determined by cytofluorometric assessment of DiOC6(3)/PI staining. The graphs depicts the percentage of viable [DiOC6(3)+] cells normalized to their respective control (DMSO) condition. Dark lines correspond to mean ± SEM (***P* < 0.01; ****P* < 0.001; Student’s *t*-test).

**Supplementary Figure 5**

**
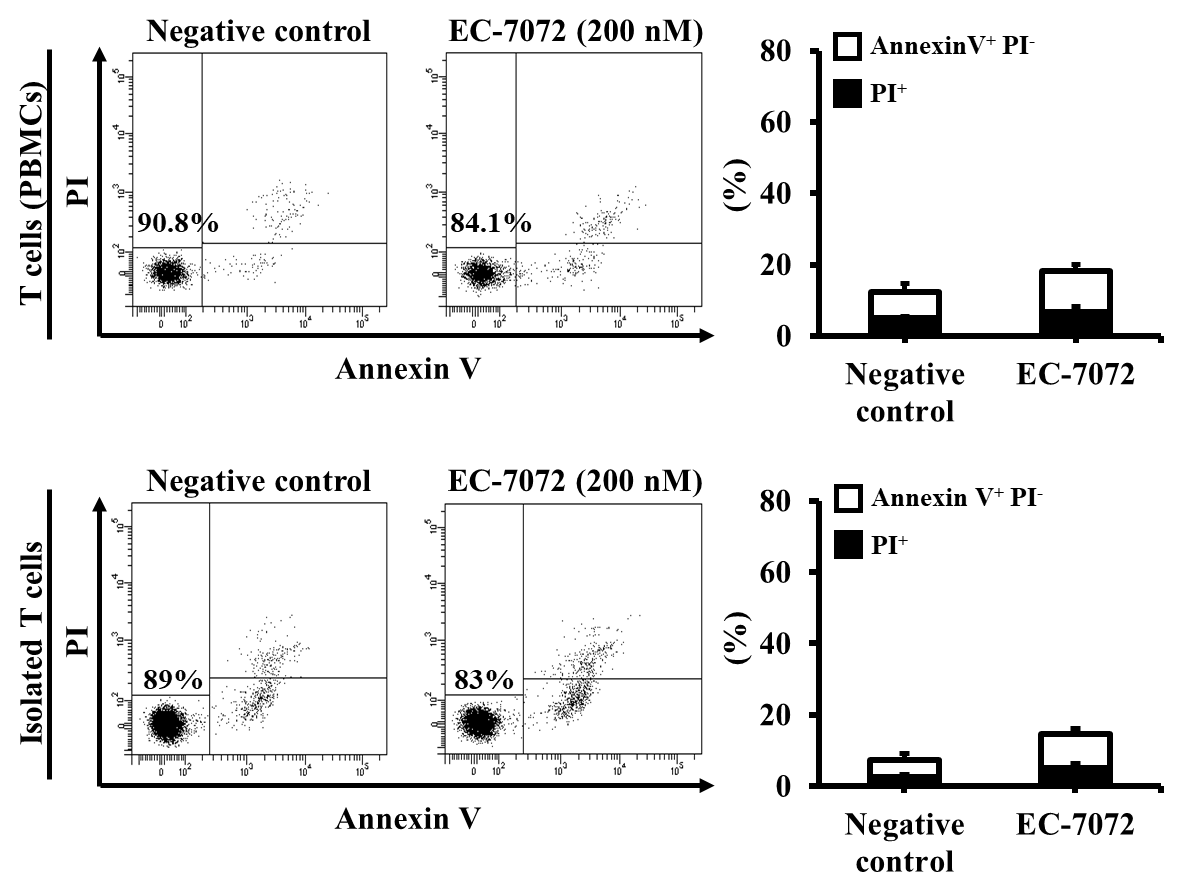
**

**Supplementary Figure 5. EC-7072 does not significantly affect the viability of isolated T cells from patients with CLL.** PBMCs and isolated T cells from patients with CLL (n = 3) were treated with EC-7072 (200 nM) for 24 hours and T cell apoptosis was examined by cytofluorometric assessment of Annexin V/PI staining. Dot plots show a representative patient and percentages within refer to viable [Annexin V^-^] cells. Graphs depict the mean percentages of apoptotic [Annexin V^+^ PI^-^] and dead [PI^+^] cells (mean ± SEM) (Student’s *t*-test).

**Supplementary Figure 6**

**
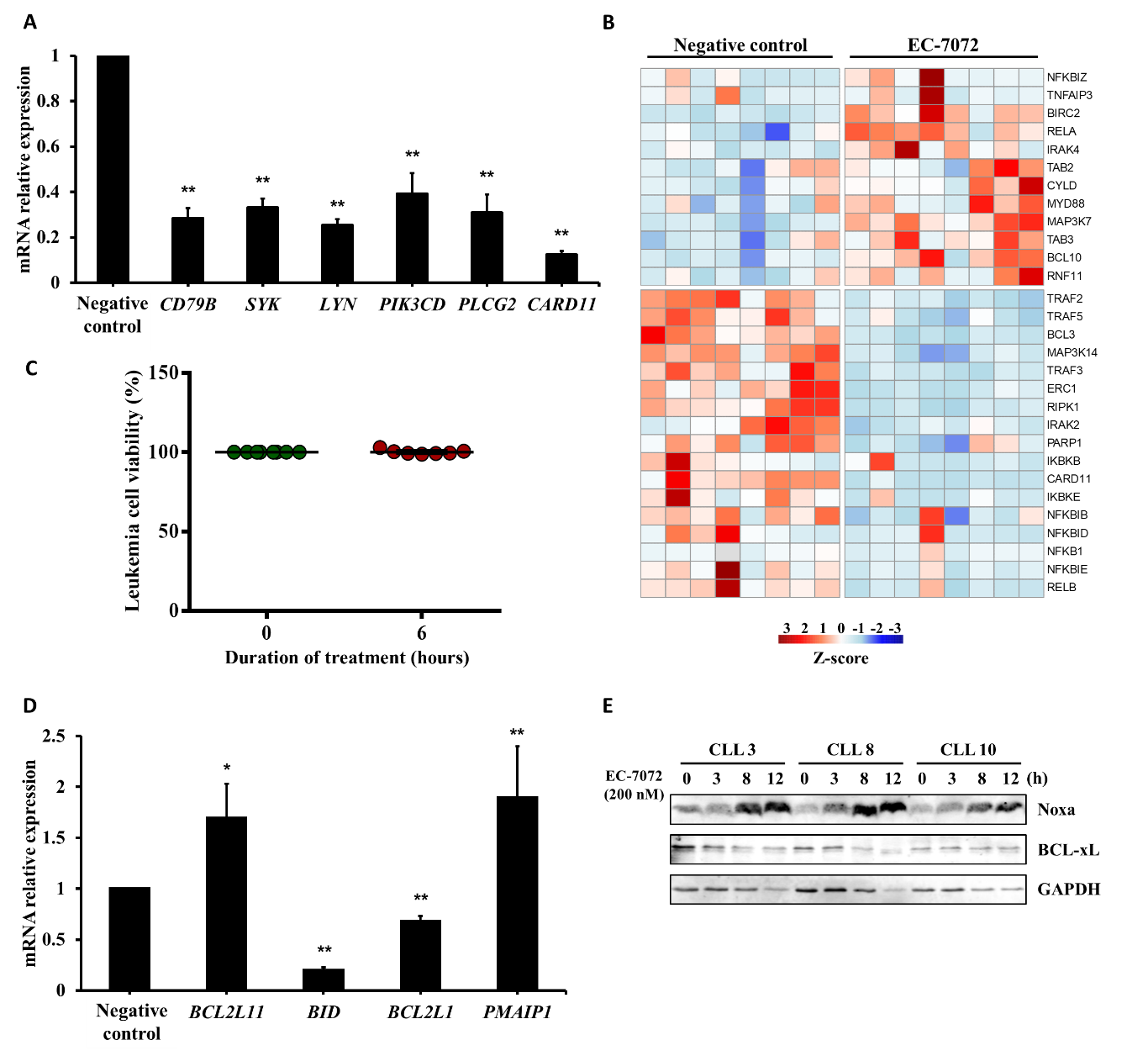
**

**Supplementary Figure 6. EC-7072 modulates the transcriptome of primary CLL cells. (A)** Expression of BCR-related genes was analyzed by qPCR in isolated CLL leukemia cells (n = 6) exposed to 200 nM EC-7072 for 6 hours (mean ± SEM). Bars depict the mean relative expression normalized to the control (DMSO) condition. **(B)** Heat map representation of significantly dysregulated genes involved in NF-κB signaling pathway in CLL cells upon EC-7072 treatment. The color scale represents the per-gene Z-score. **(C)** PBMCs from patients with CLL (n = 8) were treated with 200 nM EC-7072 for 6 hours and viability was assessed by DiOC_6_(3)/PI staining. Graph depicts the mean percentage of viable [DiOC_6_(3)^+^] cells normalized to the control (DMSO) condition for each individual patient. Dark lines represent mean ± SEM. **(D)** Expression of relevant genes that regulate apoptosis in CLL was analyzed by qPCR in isolated leukemia cells from patients with CLL (n = 6) exposed to 200 nM EC-7072 for 6 hours. Bars depict the mean relative expression to the control (DMSO) control condition (mean ± SEM). **(E)** Western blot analysis of BCL-xL and Noxa in isolated CLL cells (n = 3) treated with 200 nM EC-7072 for the indicated times. (**P* < 0.05; ***P* < 0.01; Student’s *t*-test).

**Supplementary Figure 7**

**
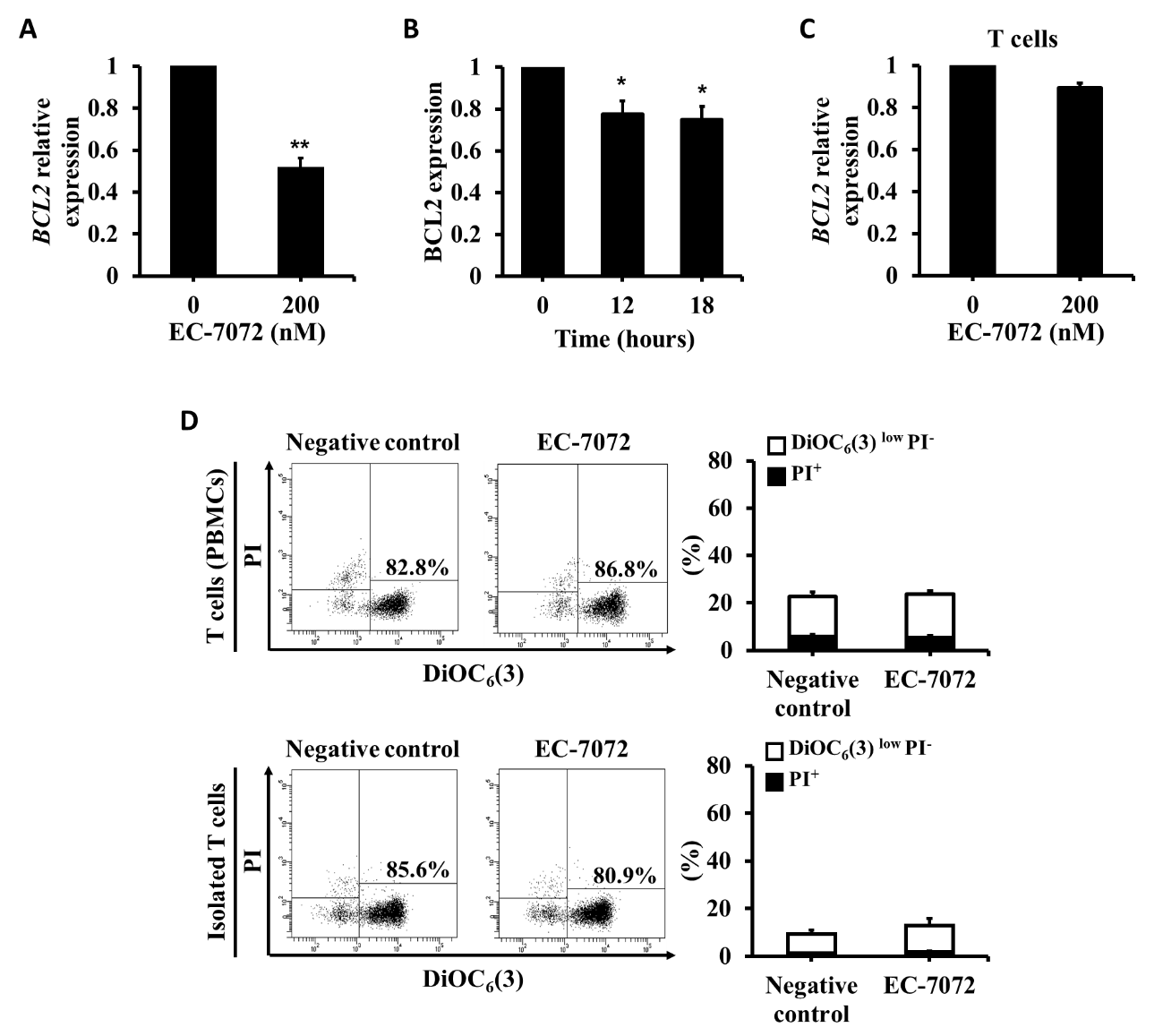
**

**Supplementary Figure 7. EC-7072 dysregulates *BCL2* expression in primary CLL cells. (A)** Isolated CLL cells (n = 6) from patients with CLL were treated with EC-7072 (200 nM) for 6 hours and total RNA was extracted. Relative expression of *BLC2* was determined by qPCR. Bars depict the mean relative expression to the control (DMSO) condition (mean ± SEM). **(B)** Isolated CLL cells (n = 6) were treated with 200 nM EC-7072 for 12 and 18 hours and BCL2 levels were evaluated by intracellular flow cytometry (mean ± SEM). **(C)** isolated T cells (n = 3) from patients with CLL were treated with EC-7072 (200 nM) for 6 hours and total RNA was extracted. Relative expression of *BLC2* was determined by qPCR. Bars depict the mean relative expression to the control (DMSO) condition (mean ± SEM). **(D)** PBMCs and isolated T cells from patients with CLL (n = 3) were treated with 200 nM EC-7072 for 24 hours. T-cell death and apoptosis were assessed by DiOC_6_(3)/PI staining. Dot plots show a representative patient and percentages within refer to viable [DiOC_6_(3)^+^] cells. Graphs depict the mean percentages of apoptotic [DiOC_6_(3)^low^ PI^-^] and dead [PI^+^] cells (mean ± SEM). (**P* < 0.05; ***P* < 0.01; Student’s *t*-test).

**Supplementary Figure 8**

**
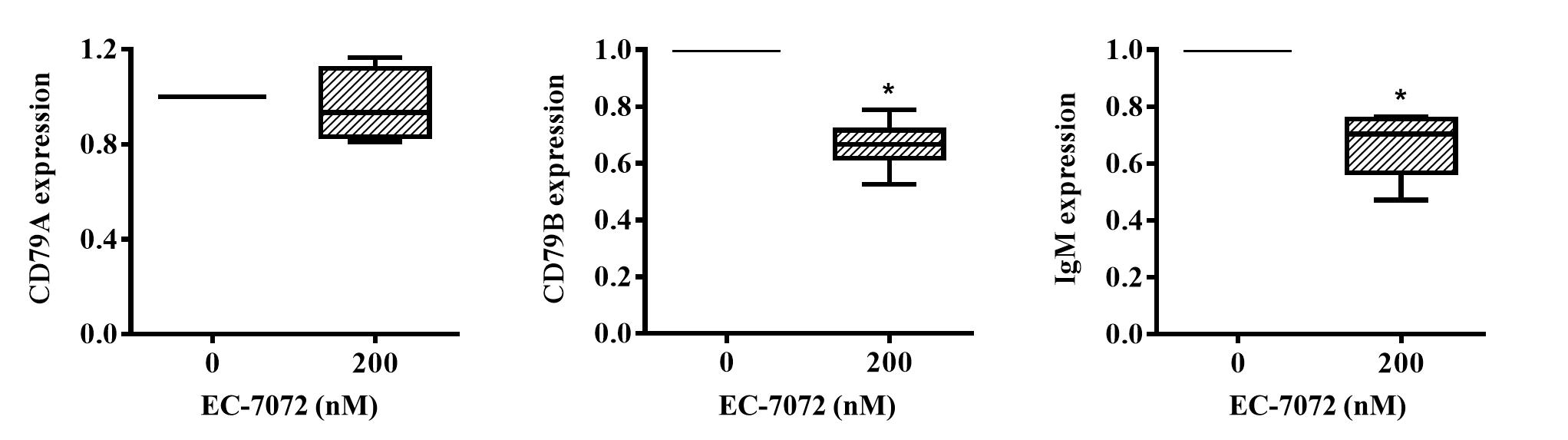
**

**Supplementary Figure 8. EC-7072 inhibits tonic BCR pathway by suppressing the expression of key signaling nodes.** PBMCs from patients with CLL were treated with 200 nM EC-7072 for 8 hours and surface expression of CD79A, CD79B and IgM in leukemia cells (n = 5-6) was assessed by flow cytometry. Boxes represent the MFI normalized to the control (DMSO) condition (**P* < 0.05; Student’s *t*-test).

**Supplementary Figure 9**

**
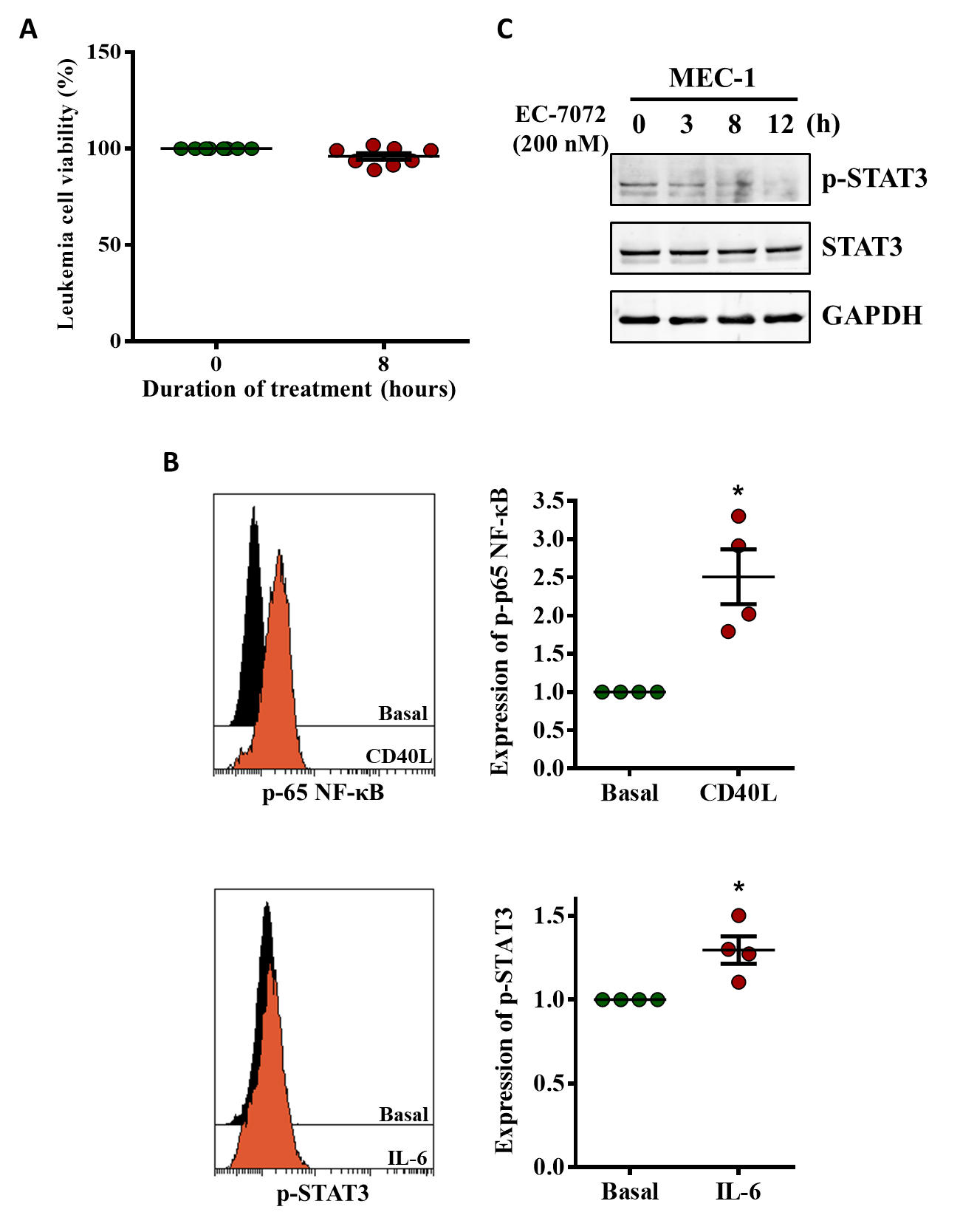
**

**Supplementary Figure 9. Activation of downstream signaling pathways with specific stimuli provide robust and reliable phosphoflow results. (A)** PBMCs from patients with CLL (n = 8) were treated with EC-7072 (200 nM) for 8 hours and viability was determined by DiOC_6_(3)/PI staining. Graph depicts the mean percentage of viable [DiOC_6_(3)^+^] cells normalized to the control (DMSO) condition for each individual patient. Dark lines represent mean ± SEM. **(B)** Phosphorylation levels of p65 NF-κB and STAT3 were detected by phosphoflow in isolated CLL cells (n = 4) stimulated with CD40L (100 ng/mL) or IL-6 (40 ng/mL) for 15 minutes respectively. Histograms show the MFI of a representative patient. Graphs represent the mean MFI normalized to the control (DMSO) condition for each individual patient and dark lines depict mean ± SEM (**P* < 0.05; Student’s *t*-test). **(C)** MEC-1 cells were treated with EC-7072 at the indicated time points and protein lysates were extracted. Phosphorylated (p)- and total STAT3 were immunoblotted.

**Supplementary Figure 10**

**
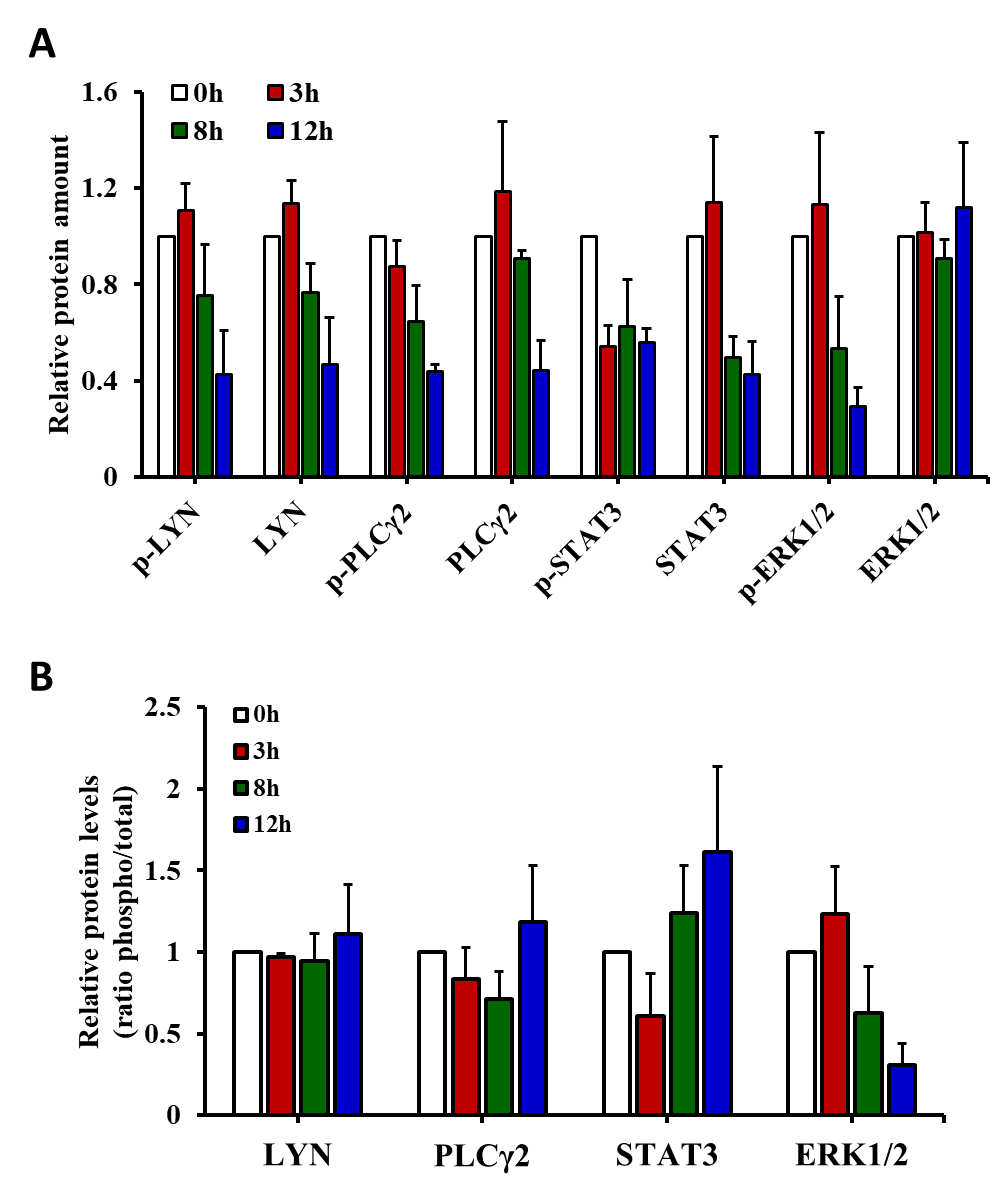
**

**Supplementary Figure 10. EC-7072 inhibits tonic BCR pathway by suppressing key signaling nodes.** Isolated leukemia cells from 3 patients with CLL were treated with 200 nM EC-7072 for 3, 8 and 12 hours. Protein lysates were immunoblotted to detect the indicated proteins. **(A)** Levels of phosphorylated and total proteins were quantified by densitometric analysis and the signal intensities were normalized to GAPDH. The graph depicts the mean value of 3 distinct patients normalized to the control (0 hours) condition. **(B)** Signal intensities corresponding to phosphorylated proteins were normalized to their respective total amount for each time point. The graph shows the mean value of the densitometry of Western blot analyses of primary CLL cells from 3 different patients normalized to the control (0 hours) condition. (Mean ± SEM).

**Supplementary Figure 11**

**
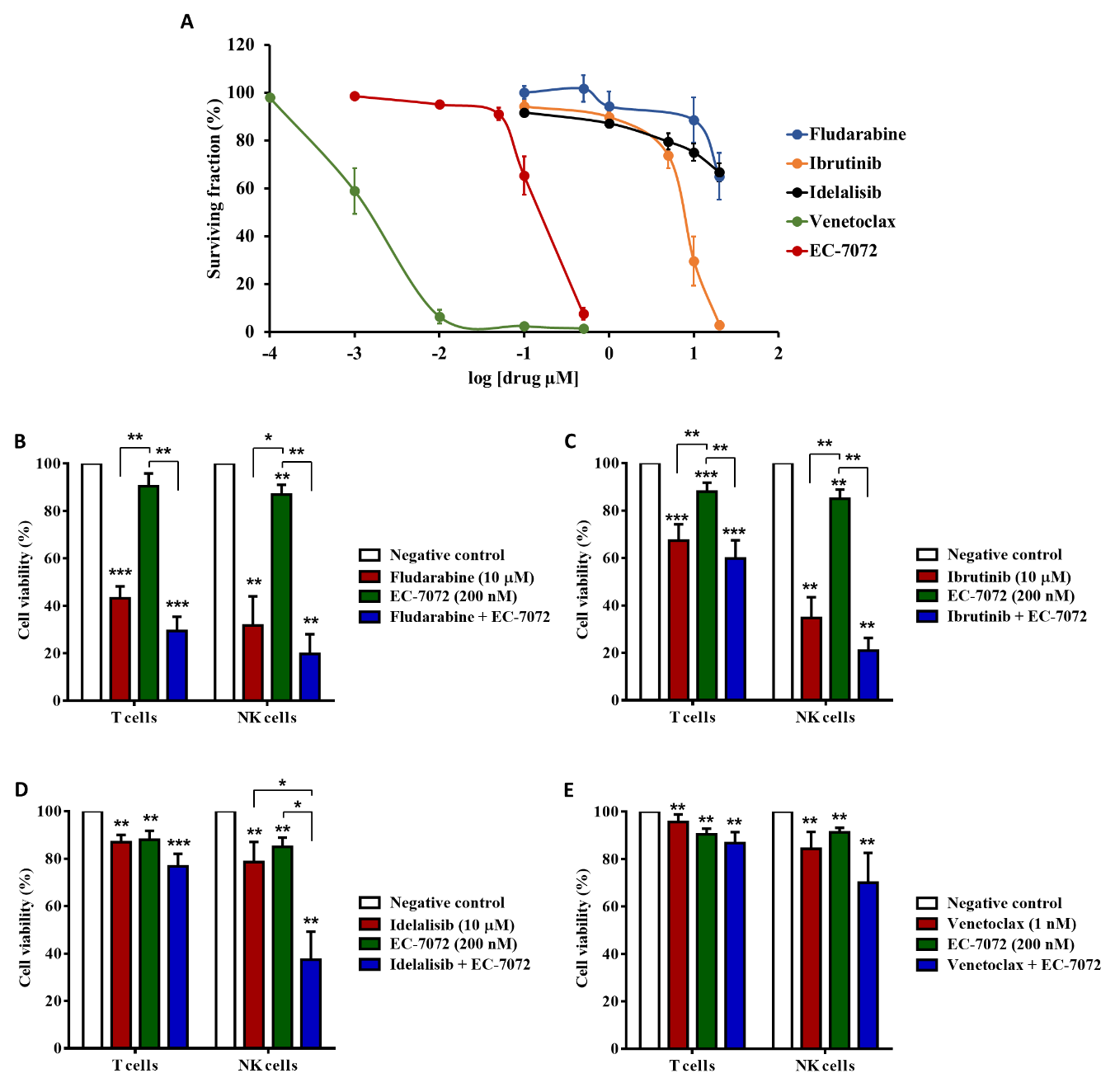
**

**Supplementary Figure 11. The antileukemic effect of EC-7072 is comparable to that of therapies routinely used in CLL. (A)** PBMCs from patients with CLL (n = 4-6) were incubated with increasing concentrations of fludarabine, venetoclax or EC-7072 for 24 hours or ibrutinib or idelalisib for 48 hours. CLL cell viability was assessed by DiOC_6_(3)/PI staining. The graph represents the percentage of viable [DiOC_6_(3)^+^] cells normalized to their respective control (DMSO) condition. **(B-E)** Cell viability of healthy immune subsets (T cells and NK cells) was evaluated in PBMCs from patients with CLL (n = 6-8) after treatment with fludarabine (10 µM) for 24 hours **(B)**; ibrutinib (10 µM) for 48 hours **(C)**; idelalisib (10 µM) for 48 hours **(D)**; or venetoclax (1 nM) for 24 hours **(E)**; and EC-7072 (200 nM) was added to the cell culture for the last 24 hours of treatment. Asterisks lacking a line underneath correspond to the significance vs the untreated control. (Mean ± SEM) (**P* < 0.05; ***P* < 0.01; ****P* < 0.001; One-way ANOVA).

**Supplementary References**

1. Cavallini C, Chignola R, Dando I, Perbellini O, Mimiola E, Lovato O, et al. Low catalase expression confers redox hypersensitivity and identifies an indolent clinical behavior in CLL. *Blood* (2018) 131(17):1942-54. doi: 10.1182/blood-2017-08-800466. PubMed PMID: 29467184.

2. Gobessi S, Laurenti L, Longo PG, Sica S, Leone G, Efremov DG. ZAP-70 enhances B-cell-receptor signaling despite absent or inefficient tyrosine kinase activation in chronic lymphocytic leukemia and lymphoma B cells. *Blood* (2007) 109(5):2032-9. doi: 10.1182/blood-2006-03-011759. PubMed PMID: 17038529.
